# The RNF220 domain nuclear factor Teyrha-Meyrha (Tey) regulates the migration and differentiation of specific visceral and somatic muscles in *Drosophila*

**DOI:** 10.1242/dev.201457

**Published:** 2023-09-14

**Authors:** Manfred Frasch, Afshan Ismat, Ingolf Reim, Jasmin Raufer

**Affiliations:** ^1^ Friedrich-Alexander-Universität Erlangen-Nürnberg, Department of Biology, Division of Developmental Biology, Staudtstrasse 5, 91058 Erlangen, Germany; ^2^ Department of Biology, University of St. Thomas, Saint Paul, MN 55105, USA

**Keywords:** Cell migration, Visceral muscle development, Somatic muscle development, RNF220, *Drosophila*

## Abstract

Development of the visceral musculature of the *Drosophila* midgut encompasses a closely coordinated sequence of migration events of cells from the trunk and caudal visceral mesoderm that underlies the formation of the stereotypic orthogonal pattern of circular and longitudinal midgut muscles. Our study focuses on the last step of migration and morphogenesis of longitudinal visceral muscle precursors and shows that these multinucleated precursors utilize dynamic filopodial extensions to migrate in dorsal and ventral directions over the forming midgut tube. The establishment of maximal dorsoventral distances from one another, and anteroposterior alignments, lead to the equidistant coverage of the midgut with longitudinal muscle fibers. We identify Teyrha-Meyhra (Tey), a tissue-specific nuclear factor related to the RNF220 domain protein family, as a crucial regulator of this process of muscle migration and morphogenesis that is further required for proper differentiation of longitudinal visceral muscles. In addition, Tey is expressed in a single somatic muscle founder cell in each hemisegment, regulates the migration of this founder cell, and is required for proper pathfinding of its developing myotube to specific myotendinous attachment sites.

## Teaser

**Summary:** Examination of the migration and morphogenesis of *Drosophila* visceral muscle precursors reveals how these multinucleated precursors form the stereotypic arrangement of longitudinal gut muscles and identifies Tey as a crucial regulator of this process.

## INTRODUCTION

In metazoans, proper organogenesis relies on precisely controlled cell migration events and dynamic cell shape changes that sculpt each organ. In most cases, different cell types participate in these events and must closely coordinate their dynamic behaviors to build a proper organ. Despite the large body of information on the regulation of cell migration and cellular morphogenesis during organ formation (Aman and Piotrowski, 2010; Scarpa and Mayor, 2016; Stock and Pauli, 2021) a full understanding of how most organs are formed is still lacking.

In *Drosophila*, one organ that has served favorably for dissecting these events is the midgut, particularly the musculature that ensheathes it. In its fully differentiated state, the musculature around the tube of endodermal cells of the midgut consists of a highly ordered arrangement of two different types of muscle fibers. One type, the circular visceral muscles, consist of elongated, binucleated syncytial cells that are oriented exactly in dorsoventral directions. As such, they form semi-circles around either side of the midgut that are attached to their contra-lateral equivalents along the dorsal and ventral midlines. The other type, the longitudinal visceral muscles, consist of multinucleated muscle fibers that extend in anteroposterior directions along the midgut tube and are oriented precisely orthogonally to the circular visceral muscles. The longitudinal muscles largely sit on top of the circular ones, although they are partially interwoven with them (Campos-Ortega and Hartenstein, 1997; Lee et al., 2005; Schröter et al., 2006). A very intriguing question is how this orthogonal network of muscle fibers assembles itself during development because the gut tube does not yet exist when the visceral muscle progenitors are born, and the progenitors of the two different visceral muscle types have completely different origins in the early mesoderm.

Both founder and fusion-competent cells of the circular visceral muscles (CiVMus) are derived from the trunk visceral mesoderm (TVM), specified as 11 segmental cell clusters by ectodermal patterning signals along the dorsal mesoderm of the trunk region (Azpiazu and Frasch, 1993; Lee et al., 2005). At the beginning of germ band retraction, these TVM clusters rearrange to form a continuous narrow band of TVM cells along the anteroposterior axis on either side of the trunk underneath the somatic mesoderm. This structure is used as a track for the migration of cells of the anterior and posterior endoderm, which ultimately meet in the middle and extend in dorsoventral directions to form the endodermal portion of the midgut tube (Azpiazu and Frasch, 1993; Reuter et al., 1993; Tepass and Hartenstein, 1994). Concurrently, each CiVMu founder cell undergoes myoblast fusion with a single fusion-competent cell from the TVM to form binucleated circular visceral muscle precursors (CiVMps), which then elongate dorsoventrally and line up into a palisade-like arrangement (San Martin et al., 2001). Continued dorsoventral elongation of the CiVMps in concert with the endoderm leads to the circular visceral musculature of the midgut.

In contrast to the TVM, which is generated along the embryonic trunk, progenitors of the longitudinal visceral muscles (LVMus) are derived from a small region at the posterior end of the early mesoderm, termed caudal visceral mesoderm (CVM) (Georgias et al., 1997; Nguyen and Xu, 1998). The CVM is specified by the bHLH transcription factor HLH54F, which becomes expressed in this region during the late blastoderm stage (Ismat et al., 2010). Cells from this cluster migrate anteriorly, thereby spreading out along the prospective midgut region (Kusch and Reuter, 1999; Lee et al., 2005). After splitting into two bilateral clusters, each cluster migrates as a collective towards the respective posterior-most cluster of the TVM on either side. Cells from these CVM clusters then stream anteriorly as a loose collective (Sun et al., 2020). This migration occurs along precise tracks at the dorsal and ventral margins of the TVM. During this migration, the CVM cells become the founder cells of the LVMus. Upon their fusion with the remaining fusion-competent myoblasts from the TVM, they form the longitudinal visceral muscle precursors (LVMps) (San Martin et al., 2001).

Some of the mechanisms guiding CVM migration have recently been uncovered (reviewed by Sun et al., 2020). The requirement for the TVM as a migration substrate has been demonstrated by genetically ablating the TVM (Zaffran et al., 2001; Reim et al., 2012). Several extracellular matrix (ECM) components have been found to promote CVM migration along the TVM. In particular, the expression of αPS2 integrin (Inflated, If) in the TVM is instrumental in the normal deposition of Nidogen in the ECM along the TVM, and mutations affecting either of these two components result in significant delays in CVM migration (Urbano et al., 2011). Mutations in lamininW subunits cause even more severe CVM migration defects, implicating lamininW as one of the key components in the ECM along the TVM to promote anterior CVM migration. αPS1 integrin (Multiple Edematous Wings, Mew), expressed in the migrating CVM cells, makes a similar contribution to the migratory efficiency as does αPS2 in the TVM (Urbano et al., 2011). Other ECM-related components, including the secreted metalloproteinase AdamTS-A, the chondroitin sulfate proteoglycan Kon-tiki and the heparan sulfate proteoglycan Trol, were also found to promote anterior CVM migration (Ismat et al., 2013; Trisnadi and Stathopoulos, 2014; Hamilton et al., 2022). In addition to facilitating direct interactions between the TVM substrate and the migrating CVM cells, it is conceivable that some of these ECM components also play roles in regulating the distribution of the FGF ligands Pyramus (Pyr) and Thisbe (Ths), which have been identified as essential signaling molecules in regulating anterior CVM migration. Importantly, a series of genetic experiments demonstrated that FGF signaling is required for proper pathfinding of migrating CVM cells, for them to remain attached to the TVM during their migration, and for securing the survival of the migrating CVM cells (Mandal et al., 2004; Kadam et al., 2012; Reim et al., 2012; Macabenta et al., 2022).

Specific migration events also take place in the somatic mesoderm and are essential for the formation of a properly patterned skeletal musculature. These involve the directed migration of somatic muscle founder cells (e.g. Dohrmann et al., 1990), followed by distinct pathfinding processes of the leading edges of the muscle precursors towards their respective myotendinous junctions (Schnorrer and Dickson, 2004). Whereas the mechanisms underlying founder cell migration are unknown, several of those involved in myotube pathfinding have been determined. Some may be similar to those described for CVM migration (e.g. laminins and Kon-tiki) (Estrada et al., 2007; Schnorrer et al., 2007; Wolfstetter and Holz, 2012; Perez-Moreno et al., 2017, 2021). Others appear specific to myotube migration, particularly the signaling components guiding the myotubes to their muscle attachment sites such as Slit, which is released from the tendon progenitors and activates Robo receptors on the surface of the extending myotubes (Schweitzer et al., 2010).

In contrast to the anterior migration of the CVM cells and their derivative longitudinal muscle founder cells, the subsequent events of the dorsal and ventral migration of the LVMps and their coordinated alignment along the length of the midgut have not been investigated extensively. Herein, we show that these muscle precursors perform these migrations as syncytia, ultimately containing approximately six nuclei, which are decorated on their surface by long and highly dynamic filopodia that extend towards their neighbors and their migration substrate. These mutual interactions, as well as the formation of anteroposterior polarities of these muscle precursors and the establishment of anterior/posterior contacts among them, are likely to be instrumental in the formation of the regular pattern of evenly spaced longitudinal muscle fibers covering the midgut. Furthermore, we identify the nuclear protein Teyrha-Meyrha (Tey), a diverged member of the RNF220 family of ubiquitin ligases, as an important factor in coordinating dorsoventral migration and proper morphogenesis of LVMps during both embryogenesis and metamorphosis. In addition to the specific expression and function of Tey in the migrating LVMps and their progenitors, we show that Tey is also expressed in founder cells and precursors of a single type of somatic muscle, termed M12 (also known as VLM1). Tey is important for the correct migration of these founder cells and proper pathfinding of the M12 muscle precursors to target their native tendon cells at the anterior and posterior segment borders.

## RESULTS

### Longitudinal visceral muscle precursors actively migrate dorsoventrally and align in parallel

**Fig. 1.**
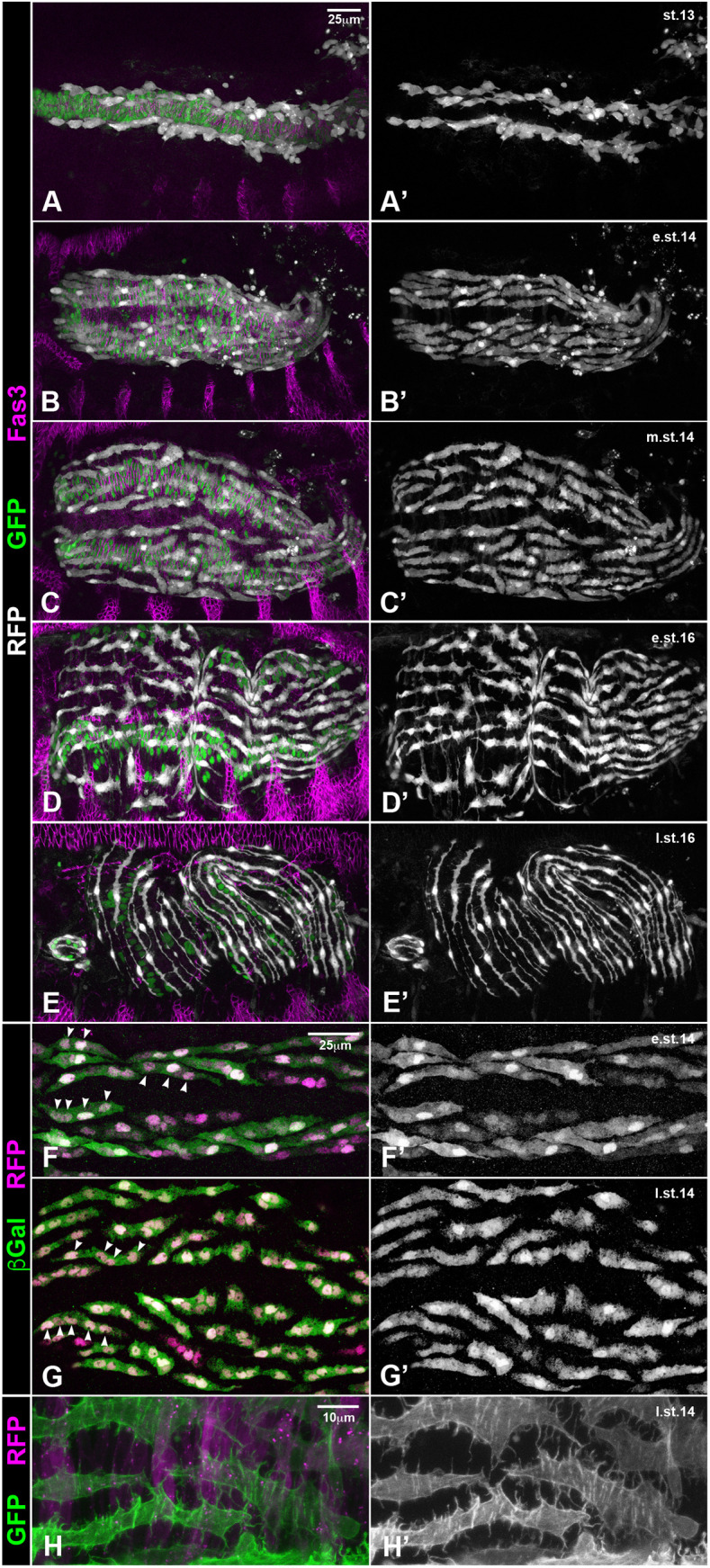
**Dorsal and ventral migration of longitudinal visceral muscle precursors.** (A-E′) Images showing the migration of LVMps in fixed embryos that express RFP in the LVMps (*HLH54Fb-cytoRFP*; white) and nuclear GFP in CiVMps (*bap3-nGFP*; green). The CiVMp cell outlines are visualized by Fasciclin 3 (Fas3) (magenta; after stage 14 also in the epidermis). (F-G′) Nuclear Histone-RFP shows that LVMus migrate as syncytia containing three to five nuclei (genotype: *UAS-His-RFP/+;tey^5053A^ UAS-lacZ/+*). Arrowheads indicate syncytial nuclei. (H,H′) Direct imaging of heat-fixed embryos (genotype: *UAS-Lifeact-GFP/bap3-RFP; tey^5053A^/TM6 Dfd-EYFP*) shows that the LVMps (green) migrate on top of the CiVMps (magenta) in the presence of pronounced filopodia. A-H show composite images; A′-H′ show LVMps only. Anterior is to the left and dorsal is up in this and other figures. e.st., early stage; l.st., late stage; m.st., mid stage. Scale bars: 25 μm (in A for A-E′; in F for F-G′); 10 μm (in H for H,H′).

Dorsal and ventral migration of the LVMps takes place in the second half of embryogenesis, in concert with the dorsoventral expansion of the CiVMps. To examine this process more closely, we marked the LVMps with *HLH54Fb-*cytoRFP (Fig. 1; depicted in white), the cell surface of the CiVMps with anti-Fasciclin3 (Fas3; depicted in magenta), and the nuclei of the CiVMps with *bap3-*nGFP (depicted in green). At stage 13, when the cells of the CVM had completed their anterior migration, the LVMps derived from them (after one or two rounds of myoblast fusion) were spread out along the dorsal and ventral margins of the narrow band of CiVMps (Fig. 1A,A′). At early stage 14, when the CiVMps elongated dorsoventrally, the LVMps elongated in anteroposterior directions and concomitantly distributed dorsally and ventrally to cover most of the expanded CiVMps, except for a central gap (Fig. 1B,B′). This process continued during the remainder of stage 14, when the central gap began to be covered as well. Notably, the elongated LVMps were largely arranged in parallel to their neighbors, although a few of them remained askew (Fig. 1C,C′). Upon dorsal and ventral closure of the midgut during stage 15, the LVMps were evenly dispersed over the midgut along with the extended CiVMps. At early stage 16, the elongated LVMps were aligned with their anterior and posterior neighbors and contacted them to form long rows along the midgut that were aligned in parallel (Fig. 1D,D′). During stage 16, this arrangement matured such that the differentiating longitudinal gut muscles formed thin muscle fibers, each one extending over much of the length of the midgut, that were distributed roughly equidistantly around the midgut and orthogonally to the circular visceral muscles (Fig. 1E,E′).

The LVMps underwent their dorsoventral migrations as syncytia, which we examined by driving (largely) cytoplasmatic *lacZ* together with nuclear histone-RFP in them. Most of the migrating LVMps already contained two to four nuclei at the beginning of this migration process during early stage 14 (Fig. 1F,F′), three to five nuclei during late stage 14 (Fig. 1G,G′), and generally six nuclei at the end of migration before lining up at stage 15 (Fig. 4J and data not shown).

Of note, the migrating LVMp syncytia featured prominent filopodia distributed over their entire cell surface. In addition to contacting the underlying CiVMps as their migration substrate, many of these filopodia extended laterally and contacted filopodia or cell bodies of neighboring LVMps (Fig. 1H,H′; see also Fig. 1C′,D′). Time-lapse videos revealed that these filopodia are highly dynamic, presumably in sensing the migration substrate and presence of neighboring cells (Movies 1, 2). These filopodia still showed dynamic activity during the parallel arrangements of differentiating LVMus at stage 16 and started disappearing at the end of this morphogenetic process at late stage 16 (Fig. 1E′; Movie 2).

### Tey is expressed in the caudal visceral mesoderm, the migrating LVMps, and the founders of somatic muscle M12

**Fig. 2.**
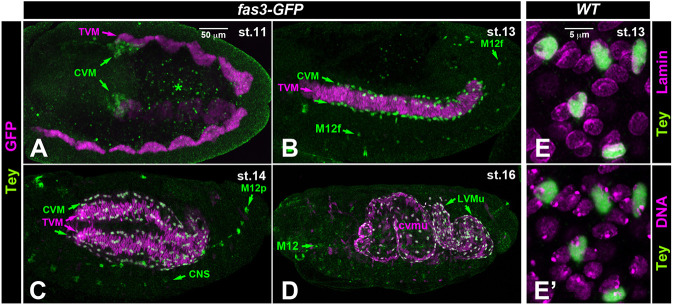
**Expression and nuclear localization of the Tey protein.** (A-D) Anti-Tey (green) and anti-GFP (magenta) staining of *Fas3-nGFP* embryos. (A) At stage 11, Tey is detected in the bilateral CVM cell clusters. (B) At stage 13, Tey is present in the nuclei of the migrating CVM cells and of single somatic muscle founder cells, M12f, within each segment. (C) At stage 14, nuclear Tey is present in the migrating LVMps and in muscle 12 precursors (M12ps). CNS expression is also evident. (D) At stage 16, nuclear Tey is present in all LVMus, but is diminished in somatic M12. (E,E′) High-magnification views confirming the nuclear localization of Tey by anti-Tey and anti-Lamin staining (E) and anti-Tey and Hoechst (DNA) staining (E′) in the same stage 13 embryo. Scale bars: 50 μm (in A for A-D); 5 μm (in E for E,F).

Previously, we presented the expression of Tey in differentiated neurons and somatic muscle M12 (Inaki et al., 2010). Here, we describe Tey expression during the migration of visceral and somatic mesodermal cells. To provide tissue context, we used *Fas3-nGFP*, which marks the TVM and CiVMus derived from it. Tey was already expressed in the CVM prior to its anterior migration; at stage 11 it was detected in the bilateral clusters of CVM cells that had migrated towards the posterior-most clusters of the TVM (Fig. 2A; see also Bae et al., 2017). Tey expression in CVM cells and their descendent LVMps persisted during their anterior and subsequent dorsoventral migration (Fig. 2B,C) and was still present in the differentiating LVMus at stage 16 (Fig. 2D). In addition, at stage 12-13, Tey expression was initiated in a single cell per hemisegment in the somatic mesoderm, which corresponded to the founder of muscle M12 (Fig. 2B). During myoblast fusion at stage 14, Tey expression was still prominent in the M12 precursors but decreased during stage 15 and thereafter (Fig. 2C,D). As already discernible in Fig. 2A-D, high-magnification views of migrating CVM cells co-stained for Tey, nuclear envelopes, and DNA confirmed that the Tey protein is strictly nuclear (Fig. 2E,E′).

### Tey is a diverged member of the RNF220 family of proteins

**Fig. 3.**
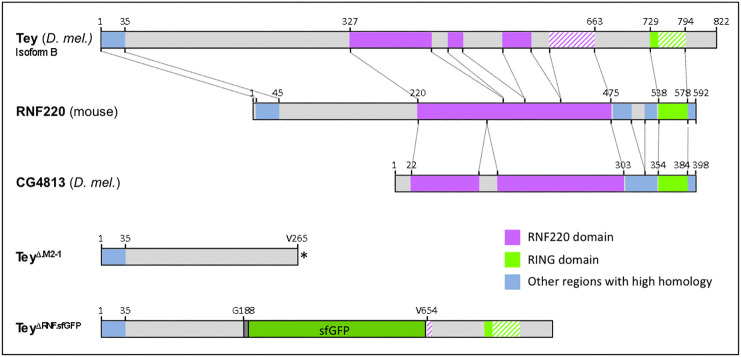
**Structure and conservation of wild-type and mutated versions of Tey and related proteins.** Top: Schematic alignments of highly conserved domains between Tey (*D. melanogaster*, *D. mel.*), RNF220 (*Mus musculus*) and CG4813p (*D. mel.*). Bottom: Schematics of the predicted Tey and Tey::sfGFP (superfolderGFP) fusion proteins translated from the respective CRISPR/Cas9-derived *tey* alleles. Asterisk indicates C-terminal stop.

Sequence alignments show that Tey is a member of the RNF220 protein family, which has representatives in all well-characterized vertebrate and most arthropod species. These proteins share a highly conserved central domain, termed the RNF220 domain, and a C-terminal RING finger domain (Fig. 3; Fig. S1). Various other stretches, such as the N terminus of Tey, are also conserved in the orthologs of other species (Fig. 3; Fig. S1). Of note, the RING finger domain of Tey, which only contains a partial RING finger motif, is highly diverged compared with those of its orthologs in other dipterans, coleopterans and vertebrates (Fig. S1). Hence, it is questionable whether the C-terminal domain of Tey is able to mediate ubiquitin ligase activity, as has been reported for mouse RNF220 (Ma et al., 2019; Song et al., 2020; Ma and Mao, 2022). However, *Drosophila* has a paralog of Tey of largely unknown function, CG4813 (recently renamed *Drosophila* RNF220; Sferra et al., 2021), which features a complete RING finger domain in addition to its RNF220 domain; in *Tenebrio*, both paralogs have a complete RING finger domain (Fig. 3; Fig. S1). The tertiary structures of *Drosophila* Tey and mouse RNF220, as predicted by AlphaFold (Senior et al., 2020), show clear structural similarities for the RNF220 domains, whereas, in accordance with the sequence alignments, no similarities are evident at their C termini (Fig. S1).

**Fig. 4.**
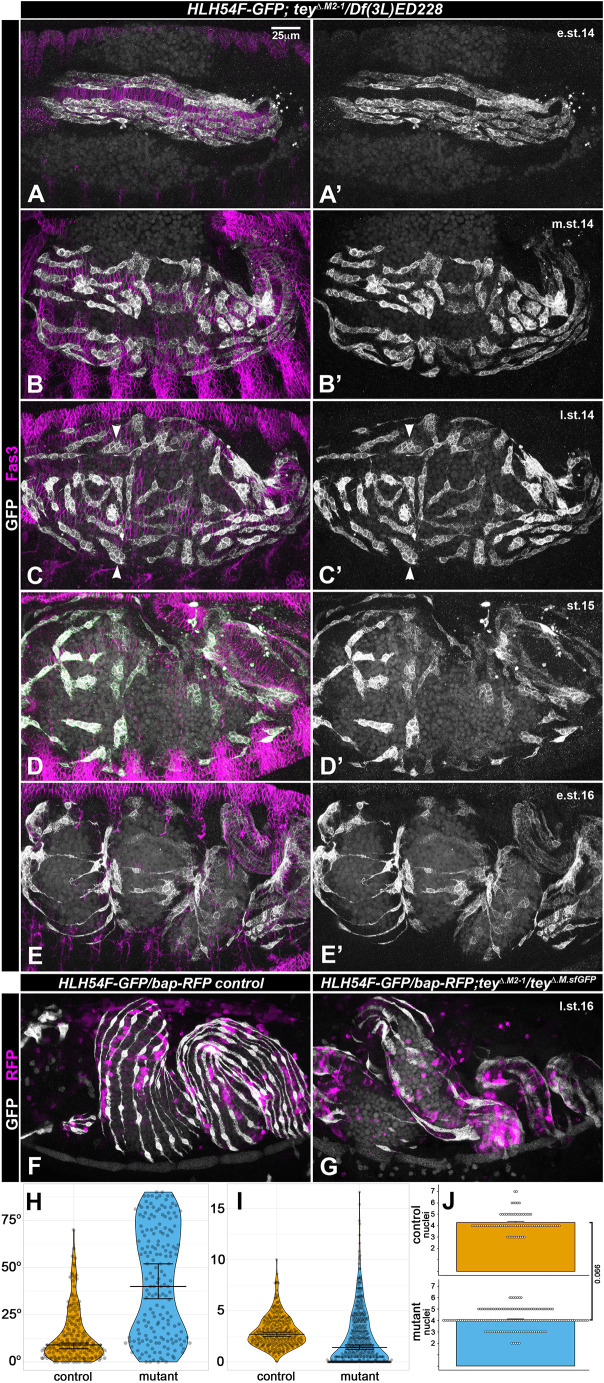
**Mis-migration of longitudinal visceral muscle precursors in *tey* mutant embryos.** (A-E′) Anti-GFP (white) and anti-Fas3 (magenta) staining of *tey* mutant embryos (genotype shown above columns). Left: dual channels; right: single GFP channel. See Fig. 1 for wild-type comparisons. (A,A′) Normal anterior LVMp migration until early stage 14. (B-C′) Mis-oriented LVMps at mid and late stage 14. (D,D′) Randomly oriented, unevenly distributed, and mis-shapen LVMps at stage 15. (E) LVMus with irregular shapes and distances at stage 16. (F) Stage 16 control embryo (either *tey^Δ.M2-1^* or *tey^ΔRNF.sfGFP^/TM6 Dfd-EYFP*) carrying *HLH54Fb-cytoRFP*/*bap3-RFP* on the second chromosome and stained for GFP (white) in the LVMus and RFP in the CiVMus. The LVMus are oriented roughly equidistantly and in parallel along the midgut. (G) Stage 16 *tey^Δ.M2-1^/tey^ΔRNF.sfGFP^* embryo with reporters and staining as in F. The LVMus are not strictly oriented in anterior-posterior directions, display variable abnormal shapes, and frequently contact their neighbors. The midgut also has areas lacking LVMus. (H) Angles of LVMPs relative to the anteroposterior axis of the midgut in control versus *tey^[P5053]^* mutant embryos at late stage 14 to stage 15. (I) Distance distributions between neighboring LVMPs at three positions each along their length in control versus *tey^[P5053]^* mutant embryos at late stage 14 to stage 15 (1 unit≈4-5 µm). Bars in H,I depict the median and the 95% confidence interval in the median. (J) Nuclear counts within LVMPs in control versus *tey* mutant embryos (*tey^m1-11^* and *tey^m2-1^* combined) at late stage 14. e.st., early stage; l.st., late stage; m.st., mid stage. Scale bars: 25 μm (in A for A-G′).

### *tey* is required for normal dorsoventral migration, including the attainment of proper orientation and spacing, of LVMus

In addition to the line *tey^5053A^*, in which a Gal4 insertion at the 5′ end of *tey* leads to undetectable expression levels of *tey* mRNA (Inaki et al., 2010) and protein (Fig. S2), we generated additional alleles using CRISPR/Cas9 to increase confidence that we are observing null phenotypes and to exclude possible effects of closely linked second site mutations. In two of these alleles, *tey^Δ.M2-1^* and *tey^Δ.M1-11^*, deletions from exon 4 to exon 8 cause the predicted native open reading frame to terminate at V^265^ and G^266^, respectively, which leads to a complete absence of the RNF220 and the diverged RING finger domains (Fig. 3, Fig. S1). A third allele, *tey^ΔRNF.sfGFP^*, produces a Tey protein in which a large central portion that includes the RNF domain is replaced with sfGFP as a tag (Fig. 3; Fig. S1; see Materials and Methods).

Until early stage 14, when the anteroposterior migration of the CVM was complete and the elongated LVMps began to disperse, we did not observe any abnormalities in *tey* mutants [Fig. 4A,A′ (compare with Fig. 1B,B′)]. However, further progression of their dorsoventral migration during mid and late stage 14, and even more so during stage 15, was highly aberrant in *tey* mutant embryos. Rather than being aligned faithfully in anteroposterior orientations and distributed evenly along the dorsoventral axis, the LVMps in *tey* mutants frequently assumed oblique or even transverse orientations, and their distances from one another were uneven. Furthermore, the shape of the syncytia became variable and abnormal during this stage as they tended to assume more squat or triangular shapes instead of the elongated spindle-like shapes [Fig. 4B-D′, arrowheads (compare with Fig. 1C,C′); Fig. 4H,I; Fig. S3B (compare with Fig. S3A)]. Their nuclei tended to be arranged in clusters instead of in single files within the LVMp syncytia [e.g. Fig. 4C,C′ (compare with Fig. 3A)]. Nuclear counts of the LVMP syncytia showed that myoblast fusion is unaffected (Fig. 4J). Although LVMp migration did reach the dorsal and ventral areas of the forming midgut tube, this migration appeared to be very uncoordinated because the LVMps were oriented in seemingly random directions. Additionally, whereas some LVMps were crowded together, other areas of the midgut remained devoid of them [Fig. 4D,D′ (compare with Fig. 1D,D′); Fig. S3D (compare with Fig. S3C); Fig. S3F (compare with Fig. S3E); Fig. S3N (compare with Fig. S3M)]. Our finding that there is no increased apoptosis in *tey* mutant LVMps suggests that the observed empty areas are a direct result of aberrant migrations rather than cell death in the absence of *tey* function [Fig. S3L (compare with Fig. S3K)]. During stage 16, some of the LVMus did manage to extend in anteroposterior directions along the gut tube, but these maintained abnormal shapes and coverage of the midgut by LVMus remained very uneven [Fig. 4E-G (compare with Fig. 1E,E′); Fig. S3D (compare with Fig. S3C); Fig. S3H (compare with Fig. S3G); Fig. S3J (compare with Fig. S3I)]. Time-lapse videos further illustrated the migration and morphogenesis defects of LVMps in the absence of *tey* function, particularly the abnormally close contacts that were frequent among migrating LVMps (Movie 3) and the resulting disorganized arrangements and morphologies of differentiated LVMus at late stage 16 (Movie 4). Filopodial extensions were still present, but we did not quantify their dynamic properties in comparison with wild-type controls.

As a result of the seemingly random migration events in mutant embryos, the final LVMp and LVMu arrangements were variable in individual embryos, but all allelic combinations tested showed essentially the same range of defects; therefore, we believe that all presented alleles are functionally null. Anti-RFP stainings of the truncated Tey::sfGFP fusion protein in *tey^ΔRNF.sfGFP^* embryos showed that the residual Tey peptide is cytoplasmic instead of nuclear (Fig. S3M-N′); the same is presumed for Tey^Δ.M2-1^ and Tey^Δ.M1-11^ (if stable). As shown in Fig. S6A-D, not only loss of *tey*, but also its forced overexpression in LVMps, disrupted their normal dorsoventral migration behavior.

To address the question of whether the product of the paralog of *tey*, *CG4813*, and/or the putative binding partner of CG4813 (and conceivably of Tey), CG13001 (Guruharsha et al., 2011; Tang et al., 2023) play a similar role in LVMP migration as does Tey, we performed RNAi experiments. However, whereas *tey*-RNAi nicely recapitulated the point mutant phenotypes [Fig. S4C,D (compare with Fig. S4A,B)], neither *CG4813*-RNAi nor *CG13001*-RNAi embryos displayed any LVMu phenotypes (Fig. S4E-J), suggesting that their gene products are not required for LVMp migration and that CG13001 does not serve as a necessary Tey binding partner during this process.

### Tey is required for proper arrangement, morphogenesis and differentiation of LVMus

**Fig. 5.**
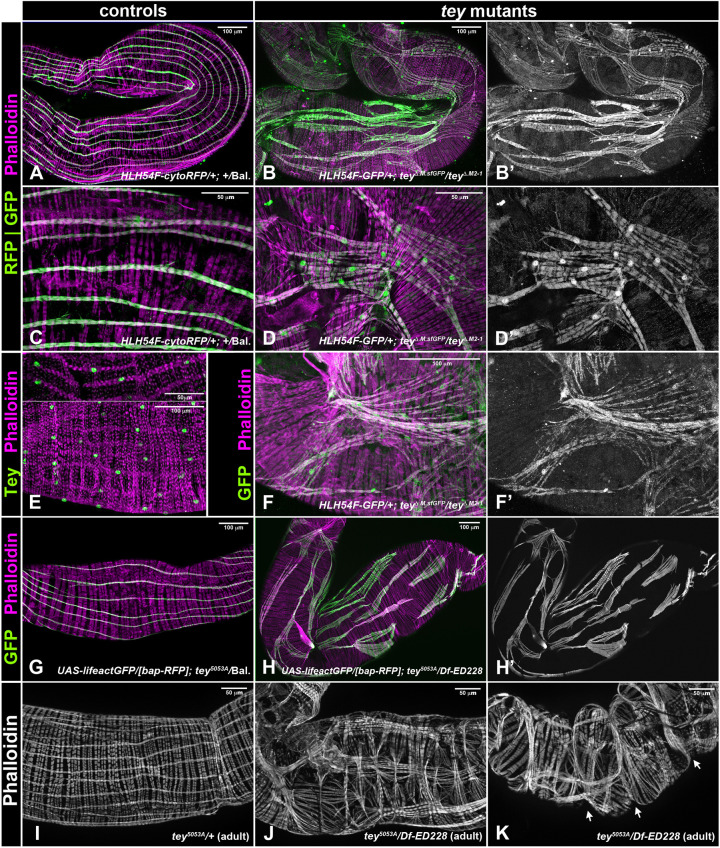
**Abnormal morphologies and differentiation of longitudinal midgut muscles in *tey* mutant third instar larvae and adults.** (A,C) Control third instar larval midguts (*+/TM6-Dfd-EYFP*) shown at two different magnifications carrying *HLH54Fb-cytoRFP* marking LVMus, and stained for RFP (green) and F-actin (magenta). The LVMus form slender muscles oriented in parallel and roughly equidistantly around the midgut. (B,B′,D,D′,F,F′) *tey* mutant third instar larval midguts (*tey^ΔRNF.sfGFP^/tey^Δ.M2-1^*) shown at three different magnifications carrying *HLH54Fb-GFP* marking LVMus, and stained for GFP (green) and F-actin (magenta). The LVMus display severe abnormalities with respect to their shapes, orientations, aberrant contacts, and myofibril alignments. (E) Third instar larval midguts stained for Tey (green) and F-actin (magenta), showing continued LVMu expression of nuclear Tey. (G-H′) Third instar larval midguts stained for GFP (green) and F-actin (magenta) (anti-RFP was negative and is not shown). (H,H′) Larval midgut from a *tey^5053A^/Df(3L)ED228* animal with reporters and staining as for the control (G), showing similar LVMu abnormalities as with the allele combination depicted above. (I) Adult control midgut (from *tey^5053A^*/*+*) stained for F-actin, showing regular spacing and orthogonal arrangements of midgut muscles. (J,K) *tey* mutant midguts from adult escapers of the genotype *tey^5053A^*/*Df(3L)ED228*, showing similar LVMu phenotypes as *tey* mutant larvae. Also, CiVMu arrangements are locally distorted and there are midgut constrictions (K, arrows).

The viability of *tey* mutant larvae (albeit decreasing until third instar) and the survival of a low percentage of adult escapers allowed us to examine the role of *tey* in the morphogenesis and differentiation of LVMus. Continued Tey expression in LVMus until at least third instar (Fig. 5E) indicates that *tey* may still be required for the continued differentiation and homeostasis of these growing muscles. Indeed, compared with the almost equidistantly spaced, thin LVMus aligned in parallel (Fig. 5A,C), the LVMus in *tey* mutant larval midguts exhibited highly disordered arrangements and aberrant shapes. Although they mostly tended to be oriented in anteroposterior directions, some were oriented obliquely or curved instead of being straight. The mutant larval LVMus were not aligned into long fibers in anteroposterior directions, showed highly variable distances to their lateral neighbors, and often touched or even crossed them [Fig. 5B,B′,D,D′,F,F′,H,H′ (compare with Fig. 5A,C,G, respectively)]. In part, these phenotypes may be due to the above-described migration defects during embryonic development. However, an additional phenotype in mutant third instar larvae concerns the morphology of the LVMus and the arrangement of their myofibrils. Specifically, the mutant LVMus were much broader, sometimes were branched or split at their ends, and their actomyosin fibrils were spaced apart along their lengths and often display frayed arrangements [Fig. 5B,B′,D,D′,F,F′,H,H′ (compare with Fig. 5A,C,G); Fig. S5B (compare with Fig. S5A)]. Nevertheless, striations were formed and showed the same range of spacing as in control larvae [e.g. Fig. 5D (compare with Fig. 5C)]. Hence, *tey* appears to continue to be required for proper morphogenesis and differentiation of the LVMus during the final embryonic and larval stages.

Because the LVMus are completely reconstituted during metamorphosis (Aghajanian et al., 2016; Schultheis and Frasch, 2023), we investigated whether *tey* is also required during this process. Indeed, the newly built LVMus from adult escaper flies showed severe abnormalities, with aberrancies that were similar to those in mutant larval midguts [Fig. 5J,K (compare with Fig. 5I)]. In addition, these abnormalities indirectly affected the arrangements of the CiVMus, which tended to be bundled together in the areas underneath the LVMus. This effect may be due to improper attachments between LVMus and CiVMus. The observed ‘accordion-like’ shape of the midgut could perhaps result from these incorrectly attached and contracting LVMus in mutant flies [Fig. 5K (compare with Fig. 5I); Fig. S5F (compare with Fig. S5E)]. In sum, we find that *tey* is required from embryonic stage 14 until early adult stages for the proper migration and differentiation of the LVMus and their precursors.

### Tey regulates the migration of somatic muscle 12 precursors

**Fig. 6.**
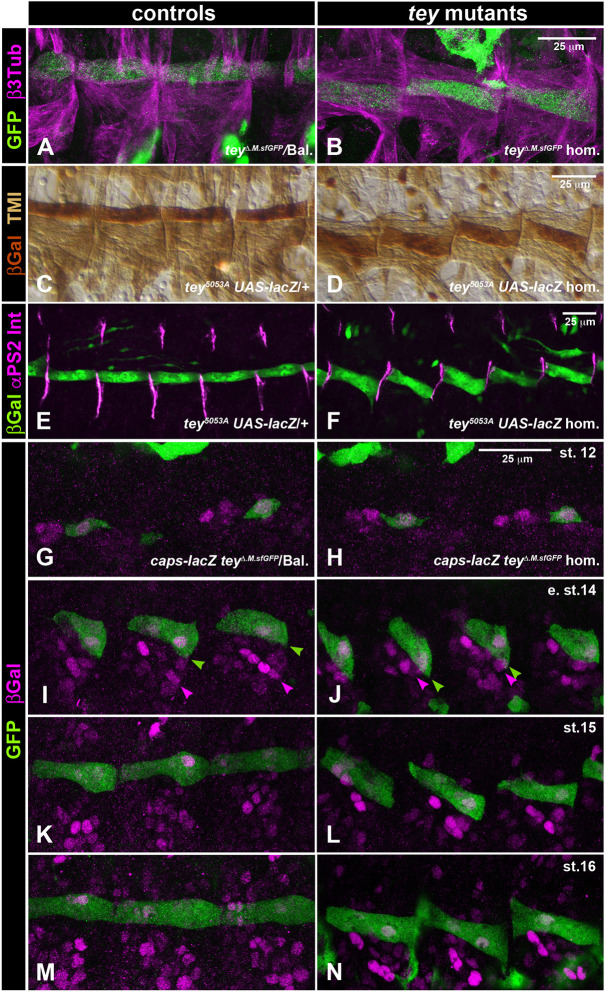
**Aberrant position and migration of somatic muscle 12 (M12) and its precursor during embryogenesis.** Left: control embryos; right: *tey* mutants with the same reporters, stainings and magnifications as the corresponding controls. (A-F) Fully differentiated muscles at stage 16. (A,B) Compared with the control (*tey^ΔRNF.sfGFP^/TM6 Dfd-EYFP*), M12 muscles in a homozygous *tey^ΔRNF.sfGFP^* mutant (stained for TeyΔRNF::GFP in green and β3-Tubulin in magenta) are shifted ventrally and display oblique orientations. (C,D) Homozygous *tey^5053A^* embryos (*tey^5053A^ UAS-lacZ*; D) display ventrally shifted M12 muscles with oblique orientations compared with controls (*tey^5053A^ UAS-lacZ/+*; C), both stained for βGal expression in M12 (dark brown) and Tropomyosin I in all muscles (TM I; light brown). (E,F) Staining of control and *tey* mutant embryos, respectively, for M12 with anti-βGal (green) and anti-αPS2 integrin (magenta) highlight the ventral shifts of M12 attachments to the segmental attachment sites in the mutant. (G-N) Developmental series showing the abnormal migration of M12ps in *tey* mutants (*caps-lacZ tey^ΔRNF.sfGFP^* homozygotes) compared with controls (*caps-lacZ tey^ΔRNF.sfGFP^/TM6 Dfd EYFP*). M12ps are marked with anti-GFP (for TeyΔRNF::sfGFP; green) and anti-βGal (for the nuclear M12 marker *caps*-*lacZ*, magenta). (G,H) At stage 12, the M12 founder cells feature identical positions and morphologies in control and mutant embryos. (I) In early stage 14 control embryos, the M12 muscle precursors arch towards their future anterior attachment sites. (J) In early stage 14 *tey* mutants, M12 migration occurs in dorsal/anterior directions and, compared with the controls, the M12p cell bodies have shifted ventrally relative to their unaffected *caps*-*lacZ*-positive neighbors (arrowheads). (K) After completing their anterior migration at stage 15, M12ps in controls establish attachments at the same dorsoventral positions as their M12p neighbors. (L) In the stage 15 *tey* mutant, the bodies of the M12ps remain shifted ventrally, with their posterior attachments showing a stronger ventral shift compared with their anterior attachments. (M) At stage 16, differentiated M12 muscles in the control form a continuous band of longitudinal muscles. (N) In a stage 16 *tey* mutant embryo, M12 muscles are arranged in a zig-zag pattern. Scale bars in right column also apply to panels on the left. Scale bar in H applies to G-N.

Tey is expressed in the founders and precursors of somatic muscle 12 (M12) (Inaki et al., 2010; Fig. 2B-D; Fig. 6A). Normally, M12 muscles (also known as VLM1) are positioned at the dorsal margin of the band of ventral longitudinal muscles (VLMs) and align at their epidermal attachment sites with their anterior and posterior M12 neighbors to form a linear row of M12 muscles along the anteroposterior axis. By contrast, in *tey* mutant embryos each M12 muscle is oriented obliquely within each segment, such that these muscles form a zig-zag pattern along the length of the abdomen (hence the name ‘Teyrha-Meyrha’, which means zig-zag in Urdu). Closer inspection with co-stainings for the other, unaffected VLMs or their epidermal muscle attachment sites showed that the anterior attachment of each M12 usually is shifted ventrally by more than the width of the muscle, whereas the posterior attachment is usually shifted by at least two muscle widths [Fig. 6B,D,F (compare with Fig. 6A,C,E)]. Thus, in addition to the orientation defects, each M12 is shifted ventrally as a whole.

To define the specific role of *tey* in M12 founder and precursor migration, we compared a developmental series of heterozygous and homozygous *tey^ΔRNF.sfGFP^* embryos that were co-labeled for Tey::sfGFP and the reporter *caps*-*lacZ*. The latter (nuclear) marker is co-expressed with Tey in founders and precursors of M12 and is also expressed in several neighboring muscle founders and precursors that can serve as positional landmarks. As seen in Fig. 6G,H, the Tey::sfGFP^+^ *caps*-*lacZ*^+^ M12 founders were shaped normally and were located at the same positions with respect to their anterior Tey::sfGFP^−^ *caps*-*lacZ*^+^ neighbors in both control and *tey* mutant embryos. In addition, *caps*-*lacZ* expression in the mutant founders showed that they maintain M12 founder cell identities in the absence of *tey* function. Upon myoblast fusion at early stage 14, it was largely the anterior end of each nascent M12 myotube that migrated towards its anterior epidermal attachment site, whereas the posterior end was already positioned close to the posterior site to which it attached during mid stage 14 (Fig. 6I). Normally, the leading edges of the migration front of muscle 12 precursors (M12ps) first moved in a dorso/anterior direction. From mid stage 14, these cells slightly bent down to contact their anterior epidermal attachment sites at the position where the posterior attachment of their anterior M12 neighbor was attached, thereby forming the linear arrangement of M12 muscles (Fig. 6I,K,M). In the absence of *tey* function, the leading edges of M12ps also migrated in a dorso/anterior direction. However, compared with the neighboring *caps*-*lacZ*^+^ muscle precursors, the entire cell bodies of M12p were positioned more ventrally, particularly at their posterior sides, in *tey* mutants compared with the controls [Fig. 6J (compare with Fig. 6I); see arrowheads). During stage 14, these muscle precursors retained their incorrect orientations and positions. During stages 15-16 they established their abnormal anterior and posterior attachment sites, leading to the zig-zag pattern of mutant M12 muscles [Fig. 6L,N (compare with Fig. 6K,M)]. We favor the interpretation that normally M12 founders and precursors migrate a short distance dorsally from their place of origin, where their leading edges can respond to pathfinding signals from their native attachment sites. However, in the absence of *tey* function they fail to undergo this dorsal migration and remain in the vicinity of the Tey^−^ Caps^+^ precursors, where they erroneously respond to pathfinding cues for their epidermal attachment from tendon cells closer to their more ventral position.

As shown in Fig. S6E-J, forced *tey* expression in *tey* mutant M12 founders and precursors rescued their migration, whereas ectopic *tey* expression in muscle founders and precursors normally lacking *tey* caused mis-migration and aberrant morphogenesis of these muscle cells.

**Fig. 7.**
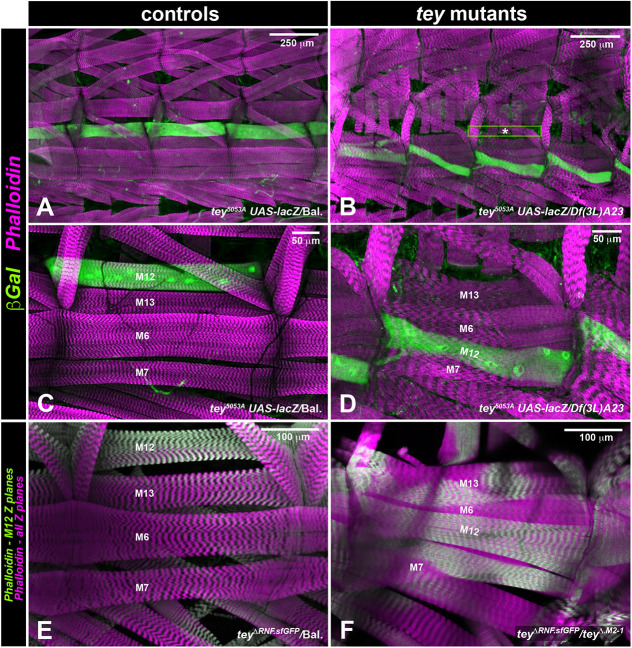
**Phenotypes of M12 muscles in *tey* mutant third instar larvae.** Left: controls; right: mutants stained for the same markers. The animal shown in B,D is at mid and all others at late third instar. (A-D) In control larvae (*tey^5053A^ UAS-lacZ/TM6 Dfd-EYFP*), M12 muscles form a longitudinal muscle band at the dorsal-most position along the ventral longitudinal muscles. In a *tey* mutant larva (*tey^5053A^ UAS-lacZ/Df(3L)A23*), M12 muscles form a zig-zag pattern. (E,F) Phalloidin-stained ventrolateral muscles of *tey* mutant third instar larva (*tey^ΔRNF.sfGFP^/tey^Δ.M2-1^*) versus control larva (*tey^ΔRNF.sfGFP^/TM6 Dfd-EYFP*). For clarity, the confocal *z*-planes that included M12 are rendered in green, whereas those containing the other muscles are rendered in magenta. Comparison of M12 position and orientation in F versus E shows that this allelic combination provokes the same M12 muscle phenotype as the one shown above.

Additional information on the mis-positioning of M12 in *tey* mutants can be gleaned from the analysis of third instar larval muscle patterns. As shown in Fig. 7B,D,F, the mutant M12 muscles were clearly shifted ventrally. Their oblique orientation is a result of the attachments at the anterior segment border to positions overlapping with the attachments of M6 (also known as VLM3) and their posterior attachments are shared with those of M7 (VLM4) (compare with Fig. 7A,C,E). Additionally, many mutant larvae showed examples of M12 muscles that display split (Fig. 7D) or broadened (Fig. 7F) attachments, indicating that muscle attachments at their more ventral positions cannot be established as consistently as at their normal positions. Similar to the gut muscle phenotypes, the observed somatic muscle phenotypes were identical for all allelic combinations tested (Fig. 7).

## DISCUSSION

### Hallmarks of late-phase migrations of longitudinal visceral muscle precursors

The main focus of our study was on the sparsely examined late phase of the migration of LVMps, which occurs after these cells have completed their anterior migration and are spread out along the length of the TVM. This culminates in an intriguing developmental process that forms an orthogonal muscle pattern ensheathing the midgut. We show that during this process these multinucleated muscle precursors perform very active migrations, with the net result being their dispersion towards the dorsal and ventral midlines of the forming gut tube. It appears that these migrating LVMps attempt to attain the largest possible distance from their respective dorsal and ventral neighbors. Because the available space for migration continuously enlarges towards the dorsal and ventral sides until the closure of the midgut tube, the mutual distances of these migrating cells increase concomitantly, such that, ultimately, the longitudinal midgut muscles are distributed in an equidistant fashion around the circumference of the midgut.

This migratory behavior is reminiscent of the processes of cellular dispersion and cellular tiling, which has been described for several other migratory cell types (reviewed by Stramer and Mayor, 2017). A known example for cellular tiling is the even dispersal of *Drosophila* hemocytes along the ventral surface of the embryo (Davis et al., 2012). Similar to hemocyte tiling and other cellular dispersion processes, it is highly likely that the dispersion of LVMus over the expanding migration substrate involves mutual repulsion among the migrating cells. This notion is consistent with the observed increased distances of regularly spaced LVMus in mutants with reduced FGF activities, in which smaller numbers of surviving LVMps are present (Reim et al., 2012). Mutual repulsion is a major component during the process of contact inhibition of locomotion (CIL), which is a well-established phenomenon of cell migration (Abercrombie, 1979; Stramer and Mayor, 2017). The distinct cellular steps and subcellular re-organizations taking place during CIL were recently described in some detail (Stramer and Mayor, 2017; Roycroft and Mayor, 2018). Because the specific processes of CIL are known to be modified in different migrating cell types, leading to distinct migration behaviors, the degree to which any of these are shared with the migrating LVMps needs to be examined.

We showed that the dorsoventral dispersion of the LVMps is accompanied by the presence of highly dynamic filopodial protrusions around the entire surface of these syncytia, which make transient contacts with filopodia or cell bodies of neighboring LVMps. For the migration of murine leukocytes, evidence has been presented suggesting that their lamellipodial and filopodial protrusions are largely functioning as ‘sensory organelles’ during migration (Leithner et al., 2016), and we propose that the filopodia of the migrating LVMps play analogous roles to mediate their mutual repulsion. Of note, the prominent presence of filopodia and the lack of lamellipodia in LVMps are features that are also seen in migrating nascent myotubes in the developing *Drosophila* testes, which represent precursors of a different type of visceral muscle (Rothenbusch-Fender et al., 2017). A recent study showed that this latter process also involves CIL-related migration behavior. Detailed analyses indicated that the dynamics of the filopodial protrusions of these myotubes requires formin-dependent formation and Rho1-linked retraction, as well as Arp2/3 dependent branching and integrin-dependent cell–matrix adhesions, all of which contribute to normal migration (Bischoff et al., 2021). However, unlike LVMps, migrating testes myotubes maintain cohesiveness and migrate collectively towards the unoccupied space, which is more similar to the behavior of other collectively migrating cells. Therefore, these two migrating types of visceral muscle precursors must differ in some important aspects of their specific molecular interplays, even though parts of the molecular dynamics described by Bischoff et al. (2021) for the filopodia of testes myotubes are likely to be active in those of the LVMps as well.

A unique and intriguing feature of the migrating LVMps is that, during their dispersion over the midgut, they initiate mutual head-to-tail contacts with their closest anterior and posterior neighbors and align to form the longitudinal visceral muscles, which extend over much of the length of the midgut. Hence, towards the end of their migration process, the proposed mutually repulsive activity initially present all around their surface must be suppressed at the anterior and posterior ends of these syncytia so that these ends can come into contact and establish stable adhesions. Although it is unclear how such a hypothesized locally restricted switch from repulsive to adhesive properties within the syncytia might be accomplished, it must rely on pre-existing anisotropies within these cells. We believe that this anisotropy is already established in the context of the previous anterior migration of the LVMps as a loose collective, when they attain anterior-posteriorly elongated spindle shapes. The elongated shape of the LVMps, which contain linear rows of nuclei, is then maintained and even increased upon continued myoblast fusion and their dorsoventral migrations, and may rely on some of the same molecular events known to organize cytoskeletal components within somatic myotubes (Schulman et al., 2015). Although some of these elongated migrating syncytia can transiently assume oblique or even transverse orientations (especially at midgut constrictions), during and after making contacts with their anterior and posterior neighbors they straighten along the anteroposterior axis and thus form the longitudinal midgut muscles. As indicated by clonal cytoplasmic markers (Klapper et al., 1998), this appears to involve subsequent inter-syncytial fusion events. Although speculative, the strict dorsoventral orientation of the CiVMPs, which serve as the migration substrate for the LVMps, could also influence the overall anteroposterior orientation and anisotropy of the migrating LVMps.

### Potential functions of Tey during visceral and somatic muscle precursor migration

In a previous study, we reported on the role of Tey in the migration and synaptic targeting of motoneuronal axons (Inaki et al., 2010). Specifically, it was shown that the expression of Tey in the somatic muscle M12 serves to repress transcription of the *Toll* gene, which encodes a transmembrane receptor that therefore becomes restricted to the ventrally adjacent longitudinal muscles M13, M6 and M7 (also known as VLM2-4). Because Toll repels synapse formation of axons from the respective motoneurons, the repression of *Toll* by Tey in M12, together with the presence of this repulsive cue in the ventrally adjacent muscles that are crossed by the migrating axons, permits the specific neuromuscular targeting of M12. These data showed that the nuclear factor Tey can function as a transcriptional regulator.

Could Tey-dependent *Toll* repression also be relevant for the functions of Tey during migration of visceral and somatic muscle precursors? In contrast to the effects of *tey* mutations, ectopic expression of *Toll* in M12 did not seem to perturb the localization and orientation of this muscle (Inaki et al., 2010), which would argue against a role of *Toll* repression by Tey in myotube attachment site targeting. However, as shown herein, the absence of *tey* function already affects the migration of M12 founder cells shortly after they are born, when the Mhc driver used for ectopically expressing Toll is not yet active. We think it is likely that mis-migration of this muscle founder in *tey* mutants is the underlying cause of the observed mis-positioning and aberrant attachment of M12, although we cannot exclude a second requirement for *tey* in the specific targeting of the attachment sites by the growing myotube. Tey-dependent *Toll* repression could still be involved in guiding M12 founder cell migration, but it seems more likely that Tey regulates genes encoding other cell-surface or cytoplasmic proteins guiding the migrating M12 founder cells. Likewise, our notion that the dispersion of the LVMps over the developing midgut is driven by mutual repulsion rather than a lack of repulsion, as during Tey-dependent axonal pathfinding, points to the involvement of different Tey downstream genes during this process. The observed phenotypes in *tey* mutants, whereby LVMps and their descendent longitudinal gut muscles fail to obey the normal distances from their lateral neighbors and often touch them, suggests that Tey could regulate the expression of some of the genes involved in CIL, as discussed above. Additionally, the observation that the LVMps are less well oriented in anteroposterior directions and generally fail to align with their anterior and posterior neighbors upon finishing their migration makes it conceivable that Tey regulates some of the as-yet-unknown activities involved in cell anisotropy and mutual adhesion of myotube tips as well. More broadly, we propose that the role of Tey in regulating the migration of these muscle precursors is analogous to the role of Slow border cells (Slbo), a cell type-specific transcription factor that is thought to regulate various migration-related target genes necessary for the migration of border cells during *Drosophila* ovary development (Montell et al., 2012).

In addition to its role in LVMp migration and alignment, *tey* is required for proper morphogenesis and differentiation of LVMus, as is most obvious from the highly abnormal shapes of these muscles and their aberrant lateral myofibril alignments in larval and adult midguts from *tey* mutants. For somatic muscles in *Drosophila*, it has been shown that lateral myofibril alignment requires mechanical tension caused by twitching during late stages of their differentiation (Weitkunat et al., 2017). Thus, the myofibril mis-alignments in LVMus from *tey* mutants could in part be a result of reduced mechanical tension resulting from the failed head-to-tail alignments of the migrating LVMps. Alternatively (or perhaps additionally), this phenotype could reflect direct functions of *tey* in LVMu differentiation that are independent of its earlier requirements in LVMps and involve yet other sets of downstream genes.

### How does Tey function at the molecular level?

As Tey lacks any DNA-binding domain and is homologous to the vertebrate E3 ubiquitin ligase RNF220, it must exert its gene regulatory functions indirectly. Like Tey, murine RNF220 is expressed in neuronal tissues of the CNS, although it is not known to be expressed in developing muscle tissues. In various neuronal contexts, murine RNF220 has been documented to mono- or poly-ubiquitylate several transcription factors, chromatin regulators, and nuclear effectors of the Wnt and sonic hedgehog pathways (reviewed by Ma and Mao, 2022). For some of these factors, RNF220-dependent ubiquitylation has activating or stabilizing effects, whereas for others it leads to their inactivation or degradation (Ma and Mao, 2022). Many of these functions require the RNF220 binding partner ZC4H2 (a zinc-finger protein) and mutations in the genes for the two proteins cause similar neuronal phenotypes (Ma and Mao, 2022). Interestingly, two independent, large-scale protein interaction screens demonstrated that the *Drosophila* ortholog of ZC4H2, CG13001, binds to the paralog of Tey*,* CG4813 (RNF220) (Guruharsha et al., 2011; Tang et al., 2023), which may indicate that some of the molecular functions of murine and *Drosophila* RNF220 proteins are shared. However, because (1) the functions of Tey described herein and by Inaki et al. (2010) concern developing muscle tissues, (2) the RING finger domain of Tey has strongly diverged, and (3) we failed to detect a requirement for *CG4813* and *CG13001* in LVMu development, the relevant molecular interactors and targets of Tey in muscle tissues are likely to be different. Their identities and functions, as well as the question of whether Tey functions as an E3 ubiquitin ligase despite its diverged C terminus, need to be addressed in future studies.

## MATERIALS AND METHODS

### *Drosophila* stocks

Offspring from *Drosophila* crosses were grown at 25°C except for *UAS/Gal4* crosses, which were developed at 29°C. The following stocks were either described in our previous publications or were obtained from the Bloomington *Drosophila* Stock Center (unless denoted otherwise): *Fas3-nGFP* (Jin et al., 2013); *HLH54Fb-GFP* (Ismat et al., 2010); *HLH54Fb-cytoRFP* (also known as P{HLH54F.LVM-RFP}16c; Hollfelder et al., 2014); *UAS-His-RFP* (Emery et al., 2005); *tey^5053A^* (also known as *tey-Gal4*; Lopez, J., 1998.11.24, personal communication to FlyBase; Inaki et al., 2010); *UAS-tey* (Inaki et al., 2010); *UAS-lacZ* (also known as *P{UAS-lacZ.B}melt^Bg4-2-4b^*; Brand and Perrimon, 1993); *UAS-Lifeact-GFP* (Hatan et al., 2011) (provided by F. Schnorrer); *bap3-RFP* (also known as *P{bap-RFP.3}7*; Reim et al., 2012); *TM6 Dfd-EYFP* (Le et al., 2006); *caps-lacZ* (Shishido et al., 1998) (provided by A. Nose); *Df(3L)A23* (Cooper et al., 2010) (provided by J. Kennison); *Df(3L)ED228* (Ryder et al., 2004); *RRHS59-lacZ* (Knirr et al., 1999); *twi-Gal4* (also known as *P{GAL4-twi.2xPE}2*; provided by G. Schubiger, University of Washington, Seattle, USA); *HN39org-1-GAL4 & S18org-1-RFP* (Schaub et al., 2012); *UAS-apoliner9* (also known as *P{w[+mC]=UAS-Apoliner}9*; Bardet et al., 2008); *nos-Gal4VP16 UAS-cas9* (BL-54593); *alphatub-piggyBacK10}M6; MKRS/TM6B,Tb* (BL-32070).

### Generation of CRISPR/Cas9 mutants for *tey*

*tey^Δ.M2-1^* and *tey^Δ.M1-11^* were generated by utilizing the system described by Port and Bullock (2016) (see also www.crisprflydesign.org) to create a UAS-inducible t::gRNA array expressing transgenes. Amplifications of three PCR fragments containing four gRNA sequences (Fig. S7) with the respective primers and pCFD6 as a template (Addgene plasmid #73915, deposited by Simon Bullock), followed by Gibson assembly with the NEBuilder® HiFi DNA Assembly Cloning Kit (New England Biolabs), were performed as described by Schaub et al. (2019). The obtained *tey*-pCFD6-4 plasmid with four *tey* gRNAs under the control of UAS sequences were injected for insertion into *AttB40* (BestGene Inc.). Homozygous transgenic lines were crossed with *nos-Gal4VP14 UAS-cas9* and balanced flies from established lines were tested by PCR and sequenced for CRISPR/Cas9-induced deletions in *tey*.

*tey^ΔRNF.sfGFP^* was generated by replacing genomic *tey* sequences between the beginning of exon 4 and the end of the RNF domain-encoding sequence with the sfGFP-3xP3-TTAA-DsRed cassette from pHD-sfGFP-ScarlessDsRed (*Drosophila* Genomics Resource Center, stock 1365; https://dgrc.bio.indiana.edu//stock/1365; RRID:DGRC_1365; donor: Kate O'Connor-Giles) (with sfGFP being in-frame) through homology-directed repair, followed by the scarless removal of the DsRed marker cassette flanked by piggyBac transposon ends via piggyBac transposase (Fig. 3).

For generating the donor plasmid, a 1.04 kb genomic 5′ homology arm, a 1.3 genomic 3′ homology arm and a 2.4 kb fragment from pHD-sfGFP-ScarlessDsRed containing the sfGFP-3xP3-TTAA-DsRed cassette were PCR-amplified with the primers shown in Fig. S7. The three fragments were joined with one another and with the 4 kb SgrAI/PstI-cut vector backbone from pHD-sfGFP-ScarlessDsRed by Gibson assembly (see above) to obtain the *tey*-sfGFP-3xP3-TTAA-DsRed donor plasmid. For generating the gRNA plasmid, amplification of fragments with the respective primers containing the gRNA sequences (see Fig. S7) followed by Gibson assembly was carried out as described above, but in this case using pCFD5 with the corresponding protocol (www.crisprflydesign.org; Addgene plasmid #73914, deposited by Simon Bullock). The donor vector and gRNA plasmid (pCFD5-4) were co-injected into *vas-Cas9* embryos (BL-55821; BestGene Inc.) and the single RFP^+^ line obtained was balanced. The DsRed-containing sequences between the TTAA-containing piggyBac recombination sites were removed via crosses with an *alphatub-piggyBac*-containing line. Sequencing of the mutated locus confirmed the intended fusion of the Tey N-terminal peptide with sfGFP in conjunction with the deletion of sequences encoding in the central portion of Tey, including the RNF domain, starting from 3L:19653900 (Figs S1, S7), as well as the scarless removal of DsRed. As designed, the sfGFP open reading frame continued in frame with ^817^V in the Tey C terminus. However, instead of continuing to the stop codon as intended, an apparent recombination error caused the sequence to continue after the second of the last codon (3L:19650976, ^821^M) with *tey* sequences starting from 3L:19651689 (^644^V) in-frame such that the diverged RING domain of Tey was fused C terminally to the mutant Tey::sfGFP fusion protein, hereafter designated as Tey^ΔRNF.sfGFP^ (Fig. 3; Fig. S7). *tey^Δ.M2-^*^1^ and Tey^ΔRNF.sfGFP^ are available at the Bloomington *Drosophila* Stock center (BL-92976 and BL-92977, respectively).

### Fluorescent antibody staining

Embryo fixation and staining procedures were performed as described by Knirr et al. (1999); larval filets as described at https://doi.org/10.6084/m9.figshare.1344809.v2); and larval and adult midguts as described by Reim et al. (2012). The following primary antibodies were used: guinea pig anti-Tey [1:800 (Inaki et al., 2010); visualized with VectaStain Elite ABC kit, Vector Laboratories, and tyramide, PerkinElmer]; rabbit anti-RFP (1:250; Rockland Immunochemicals, 600-401-215); goat anti-GFP (1:500; GeneTex, GTX26673); mouse anti-GFP (12A6; 1:100; gift from A. Schambony, FAU Erlangen-Nürnberg, Germany); chicken anti-βGal (1:200; Abcam, ab9361); mouse anti-Fas3 (1:30; Developmental Studies Hybridoma Bank, University of Iowa); mouse anti-lamin (T40; 1:30; Frasch et al., 1988); mouse anti-integrin αPS2 (1:30; CF.2C7, Developmental Studies Hybridoma Bank, University of Iowa); rabbit anti-β3 Tubulin (1:1000; gift from R. Renkawitz-Pohl, Philipps-Universität Marburg, Germany); rat anti-Tropomyosin I (1:200; MAC141, Abcam).

Secondary antibodies were generally diluted at 1:200 and included Alexa Fluor 488-conjugated donkey anti-goat (Abcam, ab150129); Alexa Fluor 555-conjugated donkey anti-rabbit (Abcam, ab150074); Alexa Fluor 488-conjugated goat anti-rabbit (Thermo Fisher Scientific, A-11008), DyLight 488- and DyLight 549-conjugated goat anti-rabbit (Thermo Fisher Scientific, 35552 and 35560); Cy3-conjugated donkey anti-guinea pig (Jackson ImmunoResearch, 706-165-148); Alexa Fluor 488- and Alexa Fluor 647-conjugated goat anti-chicken (Abcam, ab150169 and ab150171); biotinylated goat anti-guinea pig and goat anti-mouse (both 1:500; Jackson ImmunoResearch, 106-065-003 and 115-065-003). Counterstaining was carried out with Hoechst 33258 (Sigma-Aldrich); or Alexa Fluor 555 and Alexa Fluor 647 Phalloidin (Thermo Fisher Scientific).

Embryo mountings for GFP/RFP live fluorescent analysis were performed as described by Hollfelder et al. (2014).

### Statistical evaluations

Measurements of LVMp angles and distances were taken from anti-β-Galactosidase-stained embryos (genotypes *HLH54Fb-lacZ; tey^P5053^* and heterozygous controls) at late stage 14 to stage 15 (prior to the formation of the first midgut constriction). For measuring the angles, seven to eight anterior-posterior contour lines were drawn that were evenly distributed along the dorsoventral extent of each midgut image. The angle of each LVMp (drawn between its most distant ends) was measured relative to its closest contour line. Angles controls: *n*=178, median 9°, 95% CI 7-10°; angles mutants: *n*=182, median 40°, 95% CI 33.5-52°. To measure the distances between muscles, for each muscle three measurements were taken, one at its anterior end, one in the middle, and one at its posterior end, in each case measuring the distance to the nearest muscle(s). To compensate for different embryo sizes, the distance values were entered as ratios relative to the corresponding midgut length and multiplied by 100; (1 unit in Fig. 4I corresponds to ∼4-5 µm depending on the respective embryo size). Distances controls: *n*=300, median 3, 95% CI 2-3; distances mutants: *n*=474, median 1, 95% CI 1-2. Nuclear numbers were counted within LVMp syncytia of late stage 14 embryos, in which visceral myoblast fusion was in progress (genotypes *HLH54b-GFP*; *tey^M1-11^* and *HLH54b-GFP*; *tey^M2-1^*; three and seven embryos, respectively; and heterozygous controls). HLH54b-GFP is strictly cytoplasmic and the nuclei are obvious as being devoid of signals. The data were computed and plotted using SRplot (http://www.bioinformatics.com.cn/srplot).

### Microscopy

Confocal *z*-stacks of fixed specimens were acquired with a Leica SP5 II laser-scanning confocal microscope (20×/0.7 HC PL APO Glycerol, 63×/1.3 HC PL APO Glycerol). Projections of the *z*-stacks were performed with Fiji/ImageJ (v.1.52v) (Schindelin et al., 2012).

### Fly stocks and staining procedures for RNAi

Fly lines used were: *UAS-myrGFP*; *tey*-GAL4/*TM3-lacZ* (this report); *UAS-CG8780^RNAi^* (*UAS-tey^RNAi^*) (VDRC 106065); *UAS-CG4813^RNAi^* (VDRC 21950); *UAS-CG4813^RNAi^* (TRiP 61303); *UASCG13001^RNAi^* (VDRC 100808). Embryos were collected at 25°C and fixed in formaldehyde as described by Knirr et al. (1999). *tey*-GAL4::*UAS-tey^RNAi^; UAS-myrGFP* embryos were collected at 29°C. Primary antibodies used were: rabbit anti-GFP (1:5000, Invitrogen, Thermo Fisher Scientific, A-6455), rabbit anti-β-Galactosidase (1:3000, Molecular Probes, Thermo Fisher Scientific, A-11132). Secondary antibodies used were: goat anti-Rb DyLight 488 (1:500, Invitrogen, Thermo Fisher Scientific, 35552).

## Supplementary Material

10.1242/develop.201457_sup1 Supplementary information
