# Supplementary material for: The RNF220 domain nuclear factor Teyrha-Meyrha (Tey) regulates the migration and differentiation of specific visceral and somatic muscles in Drosophila
Source: Development. 2023 Sep 14;150(18):dev201457. doi: 10.1242/dev.201457 (PMC10508689; doi:10.1242/dev.201457)
Supplement: Supplementary information [file develop-150-201457-s1.pdf]

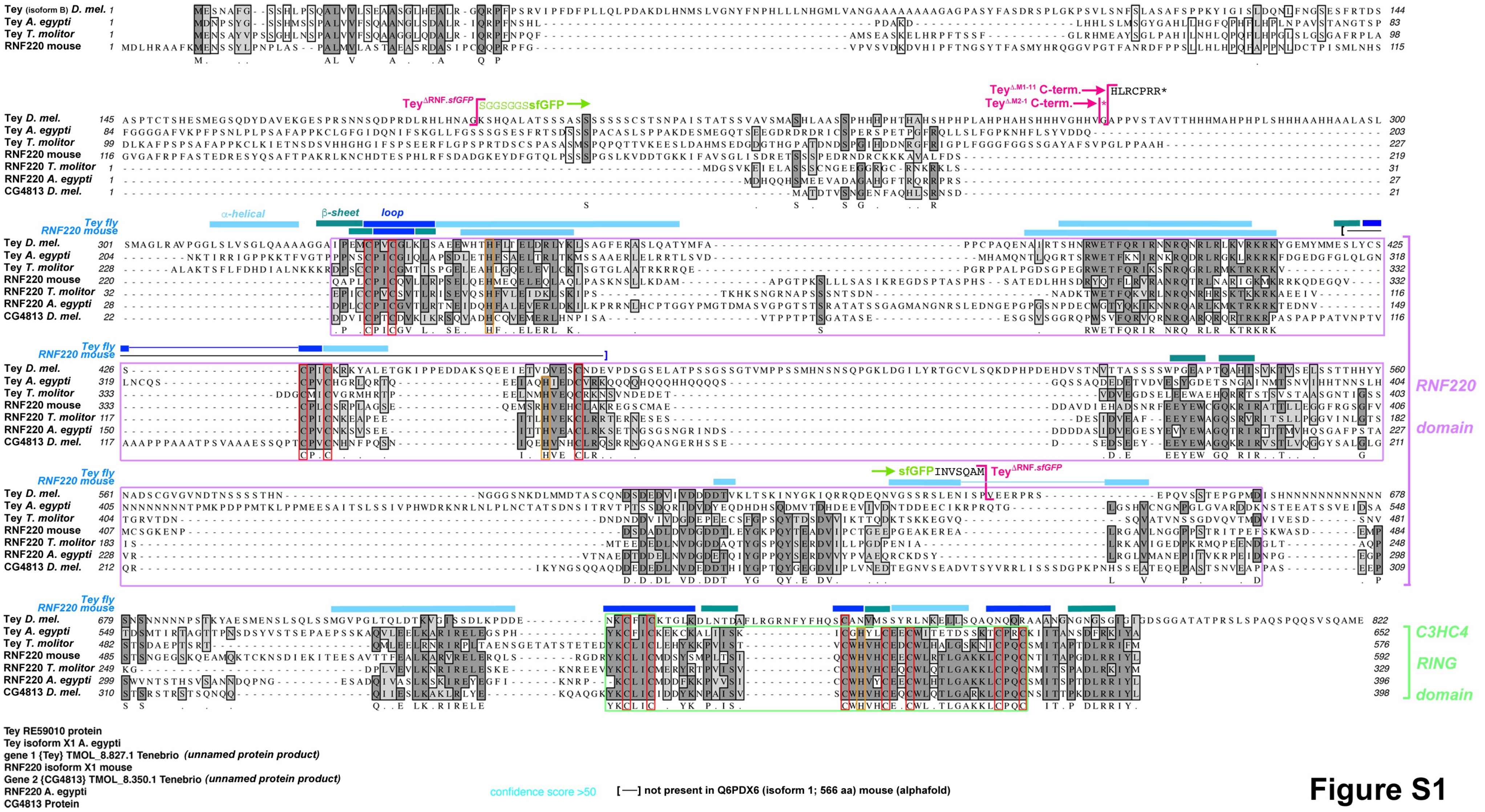

Figure S1

Fig. S1. Sequence alignments of wildtype and mutant versions of Tey from *Drosophila melanogaster* with its orthologous proteins from *Aedes egypti*, *Tenebrio molitor*, *mus musculus*, and its *D. mel.* paralog CG4813. The highly conserved RNF220 domains and RING domains are boxed in magenta and green, respectively. The bars on top of the sequences mark stretches with predicted α-helical (light blue), looped (dark blue) and β-sheet (green) conformations in fly Tey and mouse RNF220, respectively, as derived from the AlphaFold Protein Structure Database. (A stretch indicated by the thin line between brackets is absent in the mouse isoform used in AlphaFold). The C-termini of the mutant Tey$^{\Delta M2\text{-}1}$ and Tey$^{\Delta M1\text{-}11}$ proteins are depicted in red on top of the Tey (*D. mel.*) sequence. The amino acids preceding the vertical bars represent the last residues of native Tey present in the respective mutant versions, which in Tey$^{\Delta M2\text{-}1}$ is followed by a stop codon and in Tey$^{\Delta M1\text{-}11}$ by a short out-of-frame peptide sequence. Likewise for Tey$^{\Delta RNF.sfGFP}$ the transition between the N-terminal Tey sequences and a linker plus sfGFP is indicated.

Development • Supplementary information

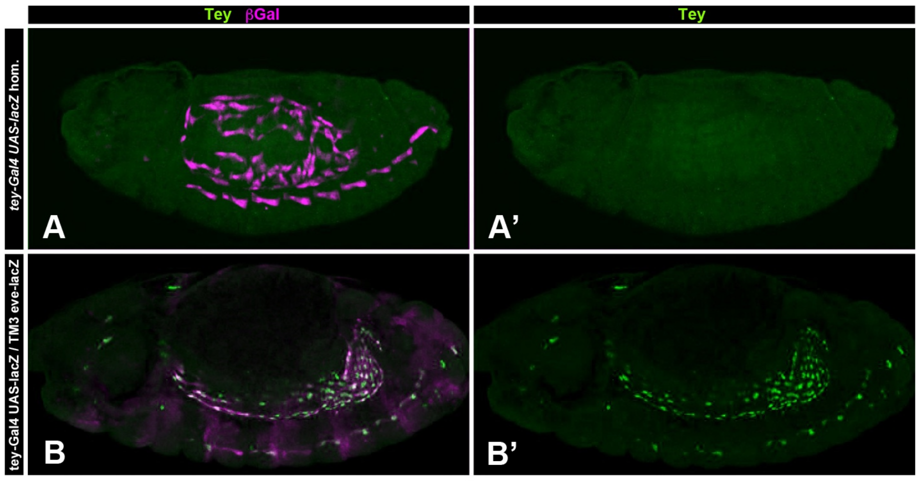

Fig. S2

Fig. S2. Lack of Tey protein expression in *tey*^{5053A} homozygous embryos and congruence of *tey*-GAL4 activity with Tey expression in heterozygotes. (A, A') Homozygous st. 14 *tey*^{5053A} *UAS-lacZ* embryo showing reporter expression in longitudinal visceral and somatic M12 muscle precursors (A) but no Tey protein in these or any other cells. (B, B') Embryo at early st. 14 *tey*^{5053A} *UAS-lacZ* in trans to *TM3 eve-LacZ* balancer showing spatial congruence of *tey*-driven reporter gene activity and nuclear Tey protein in longitudinal visceral and somatic M12 muscle precursors (as well as striped balancer-derived βGal expression).

Development • Supplementary information

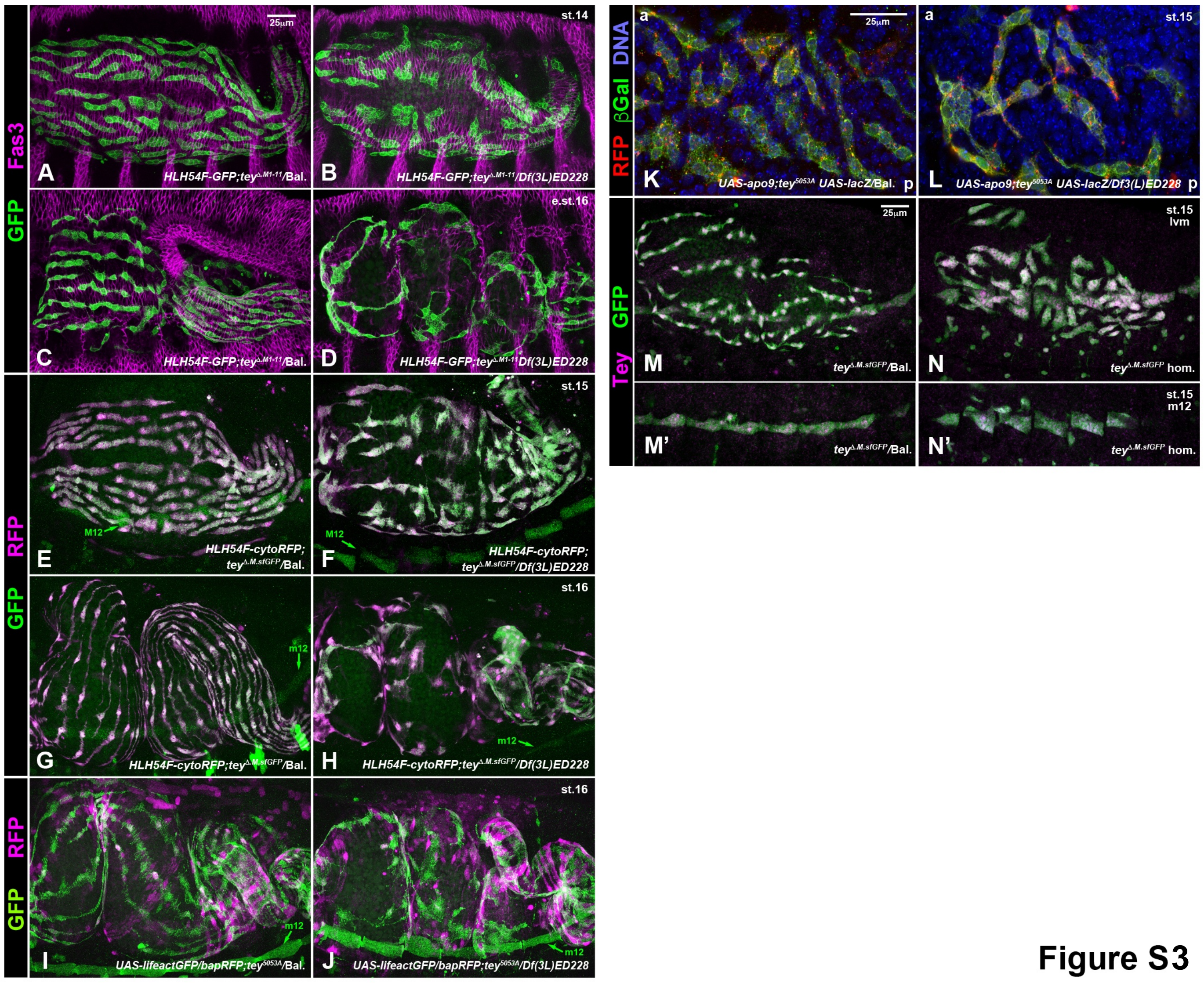

Figure S3

Fig. S3. Longitudinal visceral muscle phenotypes in embryos with various *tey* allelic combinations. Left hand column shows controls and right hand column *tey* mutants. (A – D) Stage 14 and stage 16 embryos, respectively, carrying *HLH54F-GFP* together with *tey*$^{\Delta.M1\text{-}11}$ in trans to *TM6 Dfd-EYFP* (left) or *Df(3L)ED228* (right). Anti-GFP is shown in green and anti-Fas3 in magenta. (E – H) Stage 15 and stage 16 embryos, respectively, carrying HLH54F-cyto-RFP together with teyDRNF.^{sfGFP} in trans to *TM6 Dfd-EYFP* (left) or *Df(3L)ED228* (right). Anti-GFP is shown in green and anti-RFP in magenta. (I, J) Stage 16 embryos carrying *UAS-lifeact-GFP* in trans to *bap3-RFP* on the second chromosome together with *tey*^{5053A} in trans to *TM6 Dfd-EYFP* (left) or *Df(3L)ED228* (right). Anti-GFP is shown in green and anti-RFP in magenta. (K, L) Stage 15 embryos carrying *UAS-apoliner9* on the second chromosome together with *tey*^{5053A} *UAS-lacZ* in trans to *TM6 Dfd-EYFP* (left) or *Df(3L)ED228* (right). Anti-RFP is shown in red, anti-βGal in green, and Hoechst-stained DNA in blue. As in the control (K), no nuclear RFP is detectable in the mutant (L), which argues against increased apoptosis in longitudinal visceral muscle precursors lacking *tey* function (a and p denote anterior and posterior, respectively). (M – N') Stage 15 embryos with *tey*$^{\Delta RNF.sfGFP}$ in trans to *TM6 Dfd-EYFP* (left) or *Df(3L)ED228* (right) were stained with anti-GFP for visualizing TeyΔRNF::sfGFP and with anti-Tey (magenta; ca. 50 % nuclear in the heterozygous control and largely cytoplasmatic in the mutant). For better clarity, the phenotypes in the longitudinal visceral muscle precursors were separated from those of the somatic M12 muscle precursors in the same embryos by showing the Z-planes of the former in (M, N) and the Z-planes of the latter in (M', N'). Scale bars provided in control panels also apply to the other panels with controls or mutants from the respective series.

Development • Supplementary information

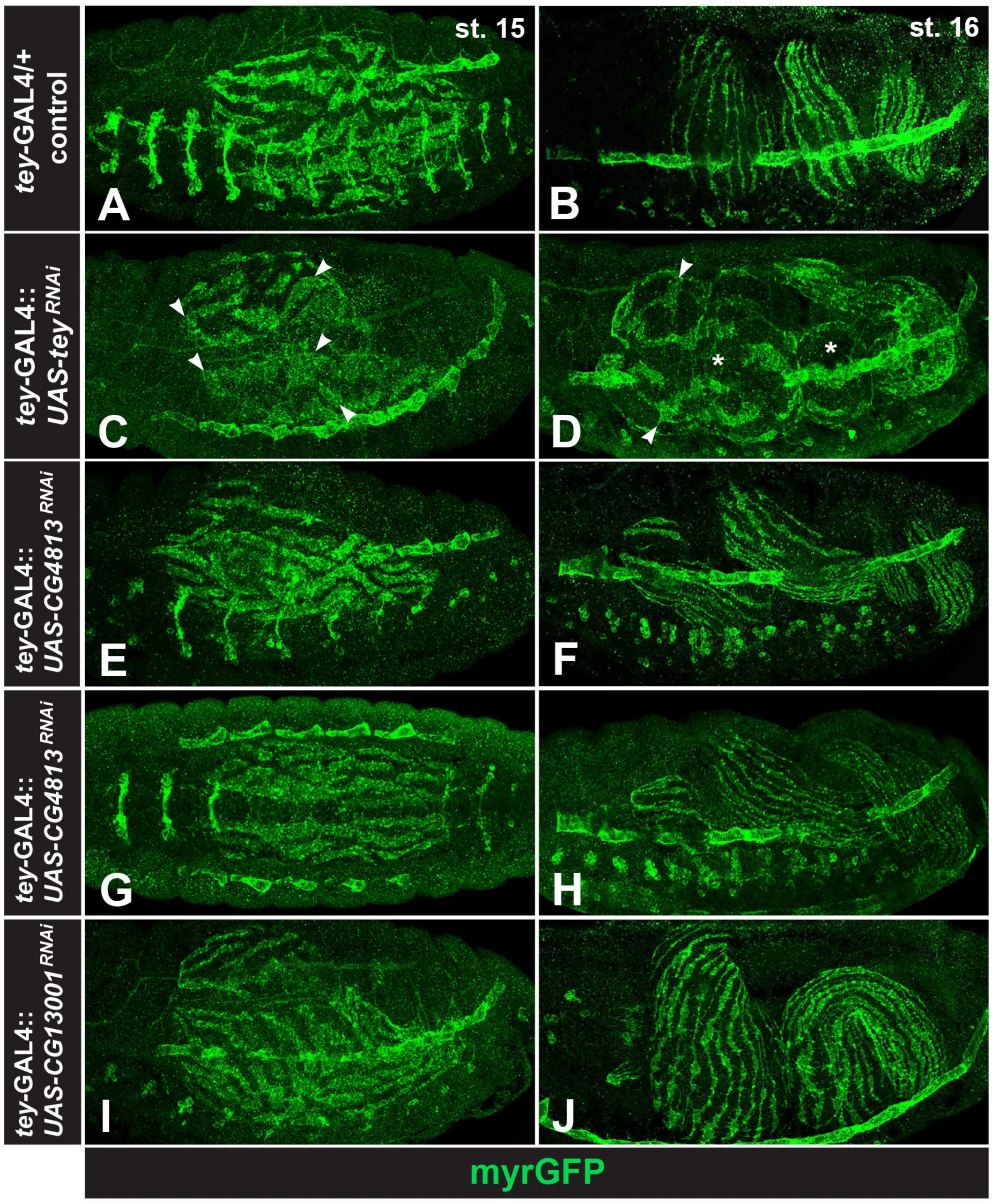

Fig. S4. Knockdowns of the *tey* paralog *CG4813* and of *CG13001* encoding a CG4813 binding partner do not elicit longitudinal visceral muscle (LVMu) precursor defects. All embryos carried *UAS-myristoylated GFP* (*myrGFP*) and *tey*-GAL4 and were stained for GFP to mark the longitudinal visceral muscle precursors (LVMp's) at stage 15 and longitudinal visceral muscles (LVMu's) at stage 16. A. Stage 15 tey-GAL4 UAS-myrGFP heterozygous (tey-GAL4 UAS-myrGFP/+) embryo showing normal development of the LVMp's. B. Stage 16 *tey-GAL4 UAS-myrGFP/+* embryo showing LVMu's that are equidistant from each other and in parallel. The milder (or absent) phenotypes in somatic muscles M12 could be explained by insufficient time for *tey* dsRNA accumulation and *tey* RNA inactivation prior to M12 founder cell migration. C. Stage 15 *tey*-GAL4::*UAS-tey^{RNAi} ; UAS-myrGFP* embryo showing aberrant arrangements and shapes of the LVMp's with disorganized rows of LVMp's (e.g., arrow heads). D. Stage 16 *tey*-GAL4::*UAS-tey^{RNAi} ; UAS-myrGFP* embryo showing mis-shapen and mis-arranged LVMu's (e.g., arrow heads) and irregular distances (asterisks). E. Stage 15 *tey*-GAL4::*UAS-CG4813^{RNAi}* (VDRC 21950)*; UAS-myrGFP* embryo showing normal development of the LVMp's. F. Stage 16 *tey*-GAL4::*UAS-CG4813^{RNAi}* (VDRC 21950)*; UAS-myrGFP* embryo showing normal LVMu's. G. Stage 15 *tey*-GAL4::*UAS-CG4813^{RNAi}* (TRiP 61303)*; UAS-myrGFP* embryo showing normal development of the LVMp's. H. Stage 16 *tey*-GAL4::*UAS-CG4813^{RNAi}* (TRiP 61303)*; UAS-myrGFP* embryo showing normal LVMu's. G. Stage 15 *tey*-GAL4::*UAS-CG13001^{RNAi} ; UAS-myrGFP* embryo showing normal development of the LVMp's. H. Stage 16 *tey*-GAL4::*UAS-CG13001^{RNAi} ; UAS-myrGFP* embryo showing normal LVMu's.

Development • Supplementary information

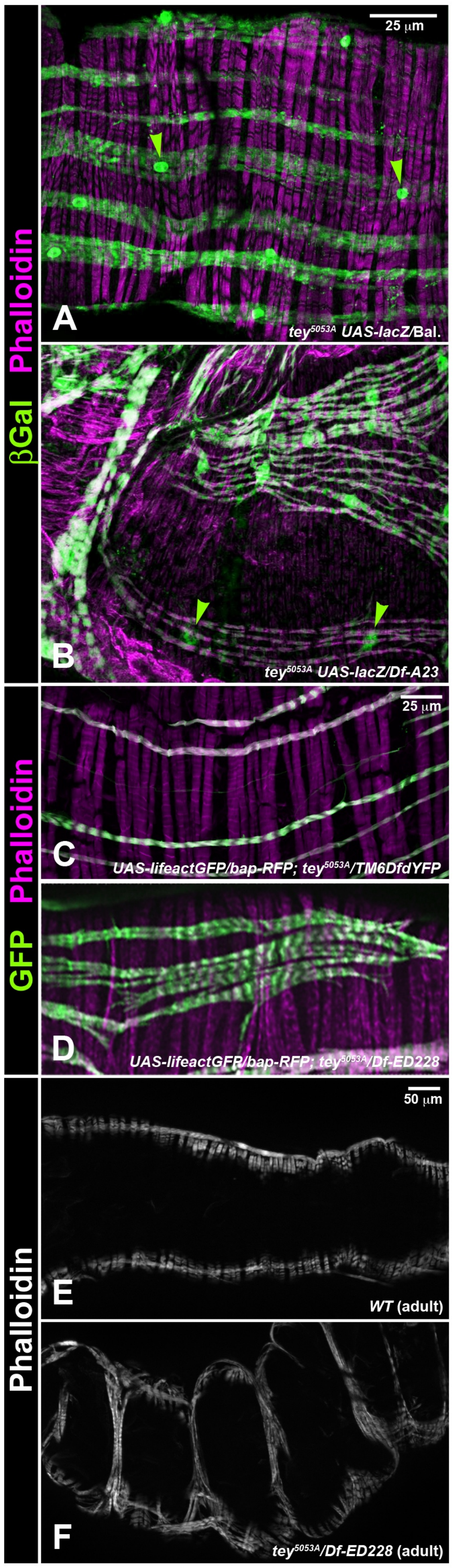

Figure S5

Fig. S5. *tey* phenotypes in midguts of 3rd instar larvae (high magnifications) and adult flies. (A, B) 3rd instar larval midguts with *tey*^{5053A} *UAS-lacZ* in trans to *TM6 Dfd-EYFP* (A) or *Df(3L)A23* (B) and stained with anti-βGal for visualizing the longitudinal visceral muscles and for F-actin (Alexa Fluor™ *555* Phalloidin) in all gut muscles. Examples of nuclei within individual longitudinal visceral muscle fibers (visible due to partially nuclear GFP) are marked by arrow heads,

Development • Supplementary information

which in the mutant allows the assignment of individual, separated actomyosin fibrils to a distinct muscle cell. (C, D) 3rd instar larval midguts with *UAS-lifeact/bap3-RFP* together with *tey^{5053A}* in trans to *TM6 Dfd-EYFP* (C) or *Df(3L)ED228* (D) and stained with anti-GFP for visualizing the sarcomeres of longitudinal visceral muscles and for F-actin (Alexa Fluor™ *555* Phalloidin) in all gut muscles (anti-RFP is omitted as it was negative in larvae). (E, F) Midguts from adult wildtype (E) and *tey^{5053A}/Df(3L)ED228* escaper fly stained for F-actin (Alexa Fluor™ *555* Phalloidin). The mutant gut (same as in Fig. 5K) has a chambered and thickened appearance, which appears to result from its excessive contraction by the aberrantly-attached longitudinal gut muscles along the a/p axis.

Development • Supplementary information

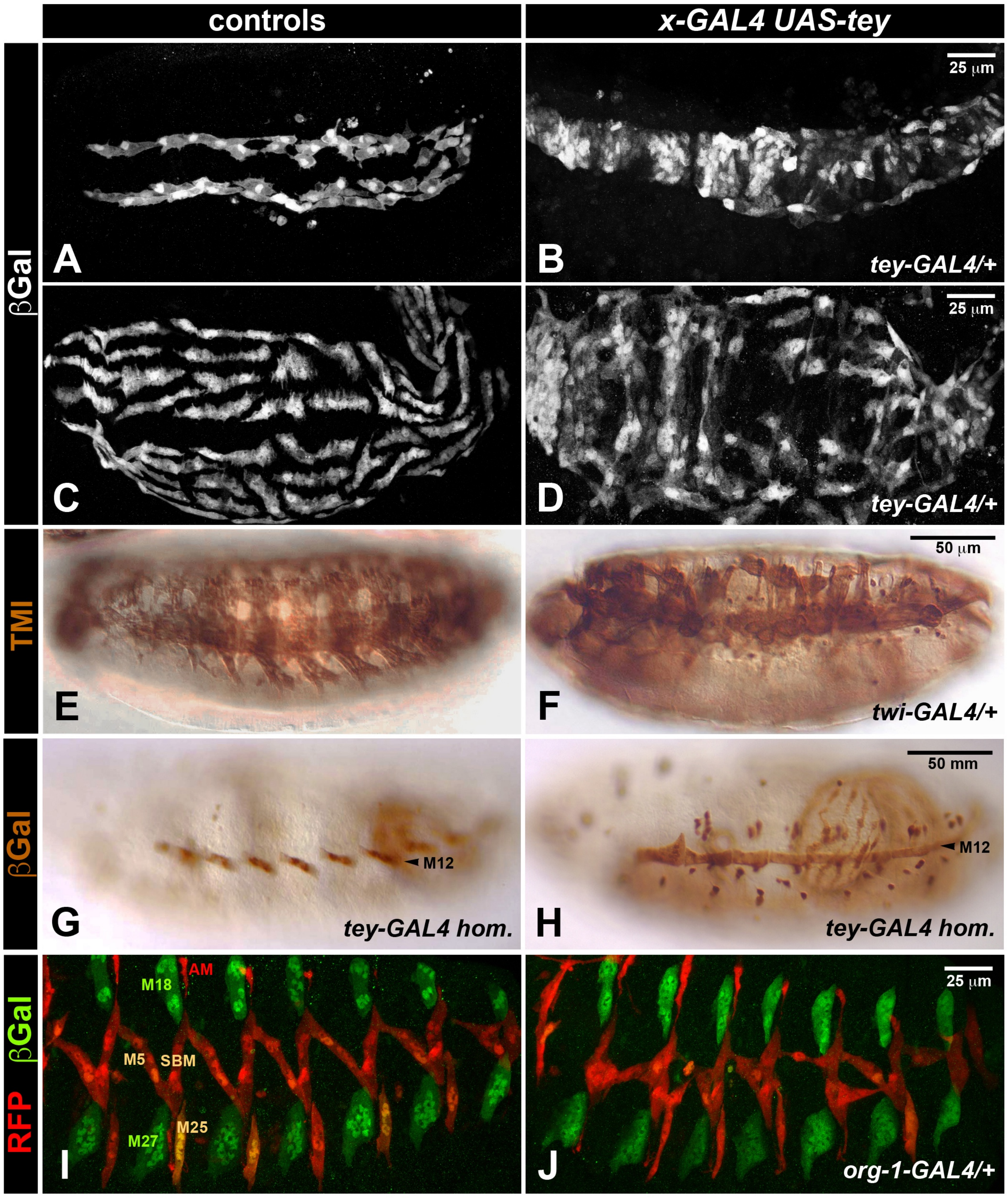

controls
x-GAL4 UAS-tey
βGal
A
B
tey-GAL4/+
25 μm
C
D
tey-GAL4/+
25 μm
TMI
E
F
twi-GAL4/+
50 μm
βGal
G
tey-GAL4 hom.
M12
H
tey-GAL4 hom.
M12
50 mm
RFP βGal
I
M18
AM
M5
SBM
M27
M25
J
org-1-GAL4/+
25 μm

Figure S6

Fig. S6. Disrupted muscle migration and morphogenesis upon forced *tey* expression in developing longitudinal visceral muscles and somatic muscles. Left hand column shows control embryos and right hand columns embryos containing both *UAS-tey* and various GAL4 drivers (denoted as *X-GAL4*, where the genotypes of X are provided on the respective panels). (A, B) As compared to the control (*HLH54Fb-lacZ UAS-tey*, stained for βGal (A)), in stage 13 embryo with *tey* overexpression within LVMp's via *tey-GAL4* (from *tey*^{5053A}) these cells prematurely spread over the entire width of the TVM. (C, D) Genotypes and staining as in (A, B). As compared to the control, stage 14 embryo with overexpression of *tey* shows abnormal orientations and shapes of LVMp's. (E, F) Somatic muscle patterns in stage 16 embryos visualized with anti-tropomyosin I show severely disrupted muscle morphologies when *twi-GAL4* is used to drive ectopic *tey* expression in the mesoderm. (G, H) Forced expression of *tey* in a homozygous *tey* mutant background (H; *UAS-tey/+; tey*^{5053A} *UAS-lacZ* hom.) rescues the zig-zag pattern seen in *tey* mutants (G; *tey*^{5053A} *UAS-lacZ* hom.) and produces almost normal M12 morphologies. (I) Stage 16 control embryo (*RRHS59-lacZ/+; HN39org-1-GAL4 S18org-1-RFP/+*) showing the normal somatic muscle patterns of the *org-1* expressing muscles M5, M25, SBM and alary muscles (AM) (anti-RFP, red) and the *slou* expressing muscles M5, M25, SBM, M18, M27 (anti-βGal, green). (J) In stage 16 embryo with ectopic expression of *tey* via *org-1-GAL4* (*RRHS59-lacZ UAS-tey/+; HN39org-1-GAL4 S18org-1-RFP/+*), the muscles with ectopic *tey* (particularly M5, M25, and SBM) display severely abnormal orientations and shapes. By contrast, the *slou*-specific muscles M18 and M27 lacking GAL4 activity are normal and can serve as landmarks. Continued expression of the *org-1*-RFP and *slou*-LacZ markers in the muscles with ectopic *tey* supports our interpretation that ectopic expression of *tey* leads to migration and morphogenesis defects in somatic muscles rather than to cell fate transformations.

Development • Supplementary information

Guide sequences for pCFD5 and pCFD6 cloning protocols (www.crisprflydesign.org)

guide seq. 1	3L:19653534.. 19653515	GCGGATTGAGCCTGGTCAGT[GGG]
guide seq. 2	3L:19653652.. 19653671	ACACCGGAGGTGCCCCCACA[TGG]
guide seq. 3	3L: 19651475.. 19651494	GTGTCCAATTGCGTTAATCC[CGG]
guide seq. 4 (rev. comp.)	3L: 19651692.. 19651711	[CCG]GTGAGATGTTCTCGAGGGAT

[genomic PAM seq.]

Primers for generating *tey*-sfGFP-3xP3-TTAA-DsRed donor plasmid:

tey 5' homology arm	
tey5-5	CCAAGGGCGAGGAGCTGTTCACCTCTCGATTGTATCTTACTCAAATGG<br>SgrAI partial
tey5-3	Reverse complement of:<br>CACACAGATCTGCATAATGCGGGGTCCGGCGGCTCAGGGGGTAGTATGGTGTCCAAG<br>*intron*/exon4 L H N A G S G G S G G S M -> sfGFP
tey 3' homology arm	
tey3-5	TGCGTCAATTTTACGCAGACTATCTTTCTAGGGTTAACGTTTCGCAGGCCATGGAGTGAGC<br>pBac *tey* stop
tey3-3	CACTAAAGGGACTAGTCCTGCAGGCTGATAGCTTGGCGAACATGTTG<br>PstI <- *oat* M
pHD-sfGFP-scarless-dsRed	
pHD5	TCAGGGGGTAGTATGGTGTCCAAG<br>S G G S M -> sfGFP
pHD3	GATAGTCTGCGTAAAATTGACGCATGCATTCTTGAAATATTGCTCTCTC<br>pBac NsiI

Blue: vector
Black: genomic

Sequence at mutated locus from C-term. of sfGFP:

```
…cgt gag gcc gcc ggc atc acc ctg ggc atg gat gag ctg tac aag att aac gtt tcg cag
… R   E   A   A   G   I   T   L   G   M   D   E   L   Y   K   I   N   V   S   Q
                          sfGFP C-term.                              Tey C-term.

gcc atg gtg gag gag cga ccg cgg tcg gag ccg cag gtg agc agc acc gaa ccc gga ccc…
 A 821M 654V  E   E   R   P   R   S   E   P   Q   V   S   S   T   E   P   G   P …
        End of RNF & diverged RING dom.
```

Fig. S7. Guide RNA sequences and primers used for generating CRISPR/Cas9 induced *tey* mutants.

Figure S 7

Development • Supplementary information

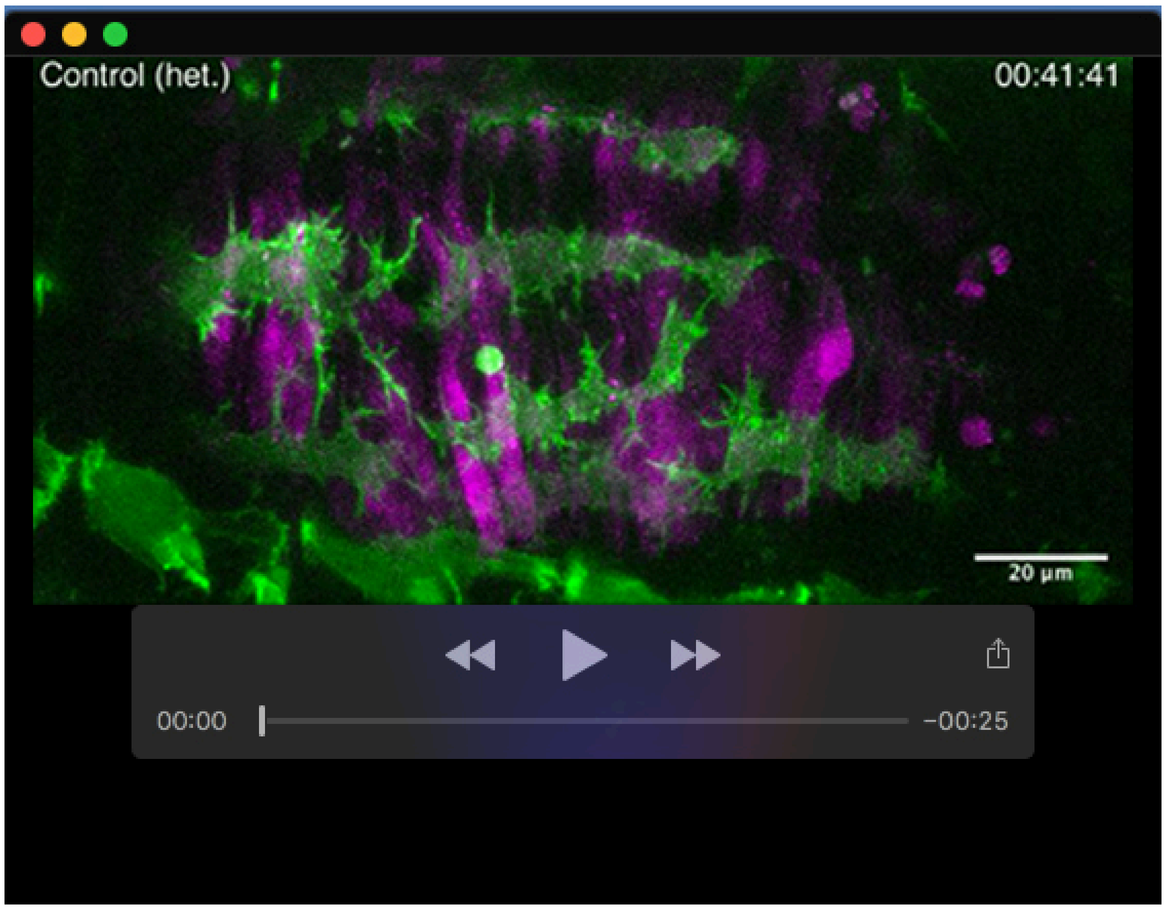

Movie 1. Time lapse movie of LVMp and LVMu migration in heterozygous *tey*^{5053A} control embryo from mid stage 14 to stage 17. LVMp's are marked with tey > *lifeact-GFP* (green) and CiVMp's with *bap3-RFP* (magenta). Open arrow heads: Examples of filopodial extensions making dynamic contacts between neighboring LVMp's and LVMu's. Closed arrow heads: Differentiating LVMu's at st. 17. M12: Somatic muscle M12.

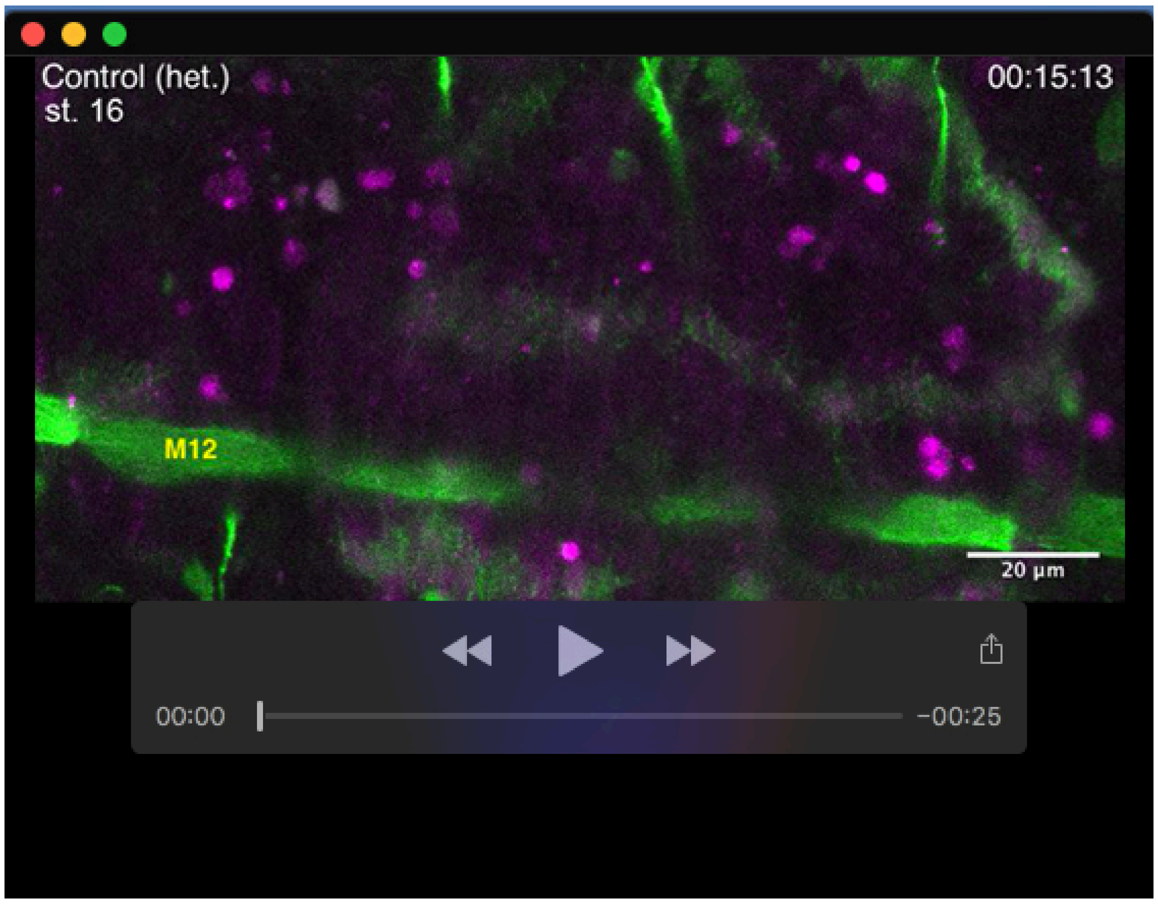

Movie 2. Time lapse movie of LVMu migration in heterozygous *tey*^{5053A} control embryo from mid stage 16 to stage 17 (focusing on late events; marked and labeled as in Movie S1).

Development • Supplementary information

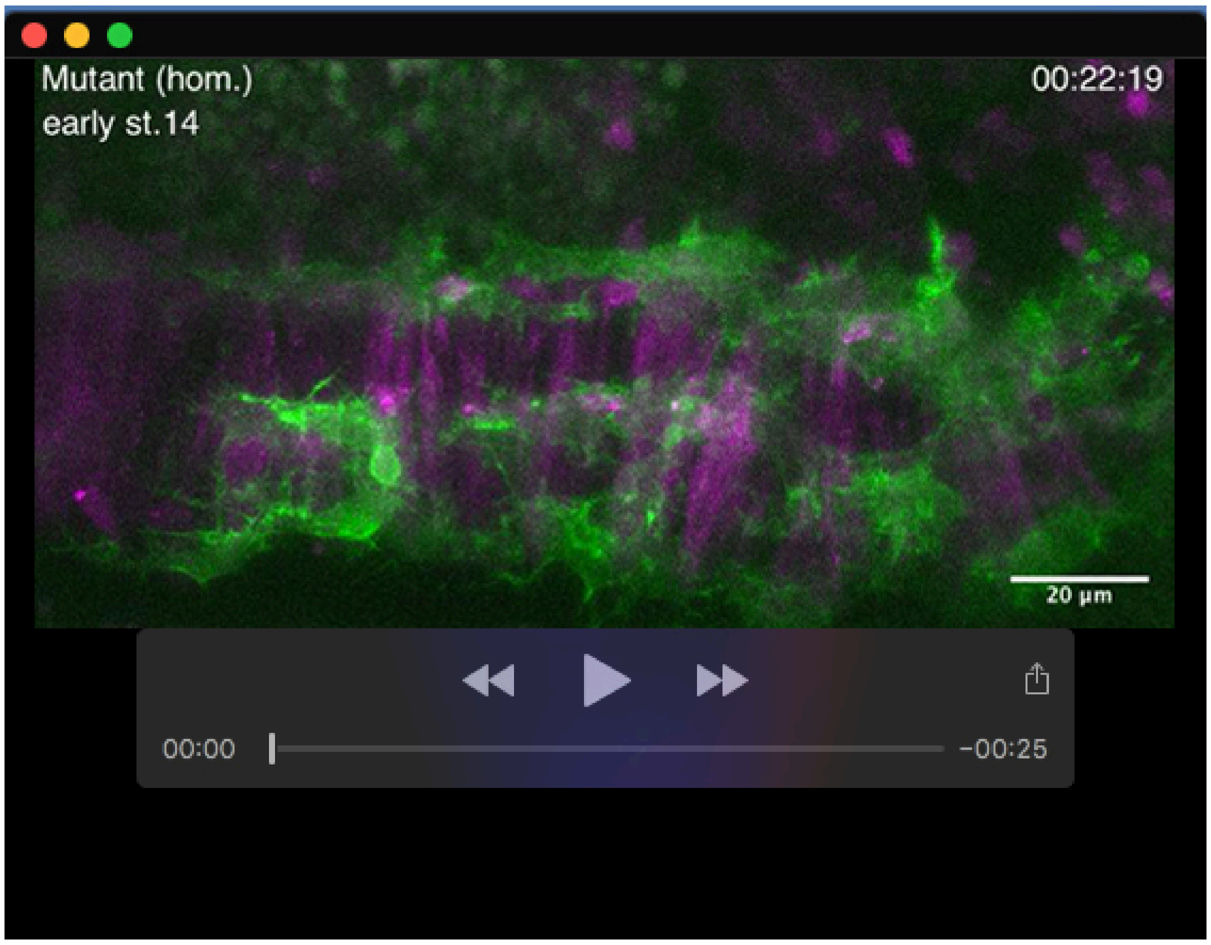

Movie 3. Time lapse movie of LVMp migration in homozygous *tey*^{5053A} mutant embryo from early stage 14 to stage 16 (marked and labeled as in Movie S1).

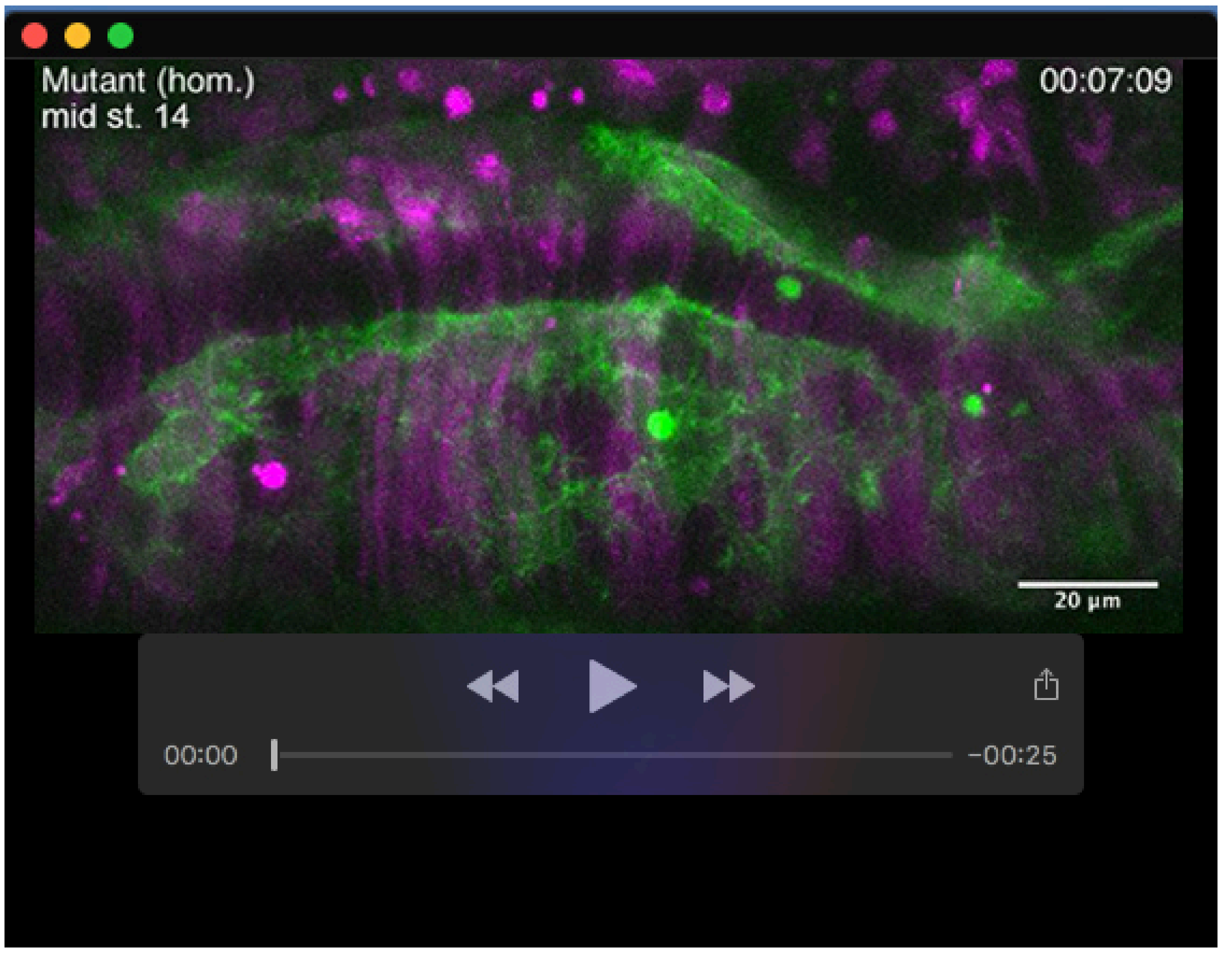

Movie 4. Time lapse movie of LVMp and LVMu migration in homozygous *tey*^{5053A} mutant embryo from mid stage 14 to stage 17 (mainly focusing on late events; marked and labeled as in Movie S1).

Development • Supplementary information
