# Supplementary material for: The RNF220 domain nuclear factor Teyrha-Meyrha (Tey) regulates the migration and differentiation of specific visceral and somatic muscles in Drosophila
Source: Development. 2023 Sep 14;150(18):dev201457. doi: 10.1242/dev.201457 (PMC10508689; doi:10.1242/dev.201457)
Supplement: Supplementary file 1 [file develop-150-201457_review_history.pdf]

The RNF220 domain nuclear factor Teyrha-Meyrha (Tey) regulates the migration and differentiation of specific visceral and somatic muscles in Drosophila

Manfred Frasch, Afshan Ismat, Ingolf Reim and Jasmin Raufer
DOI: 10.1242/dev.201457

Editor: Thomas Lecuit

Review timeline
Original submission: 13 December 2022
Editorial decision: 9 February 2023
First revision received: 4 July 2023
Accepted: 21 August 2023

Original submission

First decision letter

MS ID#: DEVELOP/2022/201457

MS TITLE: The RNF220 domain nuclear factor Teyrha-Meyrha (Tey) regulates the migration and differentiation of specific visceral and somatic muscles in Drosophila

AUTHORS: Manfred Frasch, Afshan Ismat, Ingolf Reim, and Jasmin Raufer

I sincerely apologize for the long delay in getting back to you with a decision. I have now received all the referees reports on the above manuscript, and have reached a decision. The referees' comments are appended below, or you can access them online: please go to BenchPress and click on the 'Manuscripts with Decisions' queue in the Author Area.

The evaluation of two of the reviewers is very positive and they make useful suggestions to further improve the manuscript. In light of this, we would like to publish a revised manuscript in Development, provided that the referees' comments can be satisfactorily addressed. Please attend to all of the reviewers' comments in your revised manuscript and detail them in your point-by-point response. If you do not agree with any of their criticisms or suggestions explain clearly why this is so. If it would be helpful, you are welcome to contact us to discuss your revision in greater detail. Please send us a point-by-point response indicating your plans for addressing the referee’s comments, and we will look over this and provide further guidance.

Reviewer 1

Advance summary and potential significance to field

Cell migration plays a key role in a wide variety of biological phenomena that take place during both embryogenesis and in the adult organism. In this work, the authors have used the migration of the precursor of the longitudinal visceral muscles (LVMu´s), which extend in an anterior-posterior direction along the midgut tube, to identify new molecular and cellular mechanisms regulating cell migration during organogenesis. The LVMu´s progenitors derive from the caudal visceral mesoderm, a small region at the posterior end of the early mesoderm and migrate to cover the midgut tube. Previous studies have already described the first steps of the migration of the precursor of the

© 2023. Published by The Company of Biologists under the terms of the Creative Commons Attribution License (https://creativecommons.org/licenses/by/4.0/).

LVMu´s, when they migrate anteriorly along the dorsal and ventral margins of the trunk visceral mesoderm. This work extends these studies focusing on the subsequent events of dorsal and ventral migration and their coordinated alignment along the length of the midgut. The authors provide convincing evidence that LVMu´s precursors perform this migratory phase as syncytia, which extend highly dynamic filopodia. They then identify the nuclear protein Teyrha Meyrha (Tey), a diverged member of the RNF220 family of ubiquitin ligases, as a key regulator of this dorso-ventral migration. In addition, by generating tey mutants, they show that Tey is also required for proper LVMu´s morphogenesis, both during embryogenesis and metamorphosis. Finally, they report that Tey is also expressed in and required for the correct migration of a single somatic muscle type, muscle M12.

The manuscript as a whole is written in clear and reader-friendly fashion. In addition, although there is some data missing, especially with respect to quantification and interpretation of some of the phenotypes (see below), most experiments are satisfactorily performed, well-described and properly presented. However, I find the work somewhat limited in scope, as the study- in its present state- fails to address in detail the origin of the phenotypes and also the mechanisms underlying the central molecular notion it puts forward, namely, the manner by which tey could be regulating cell movement. Therefore, I do not believe it represents an advance in the field significant enough as to merit publication in Development.

Comments for the author

My major comments for revising the manuscript for extending the scope of the sutyd are:
1. The authors claim that the LVMp's in tey mutants frequently adopt oblique or even transverse orientations, and their distances from one another are uneven. How frequent is this phenotype? I think the authors should quantify these two phenotypes, wrong orientation and aberrant distance between each other.
2. The authors state that filopodia are still present in the mutant LVMp's but they have not quantified their dynamic properties. I believe that analysing in more detail some aspects of the dynamic behaviour of this filopodia in both mutant and forced overexpression contexts may help them to get some insight into possible mechanisms by which Tey might regulate muscle cell migration.
3. The authors claim that the nuclei tend to be arranged in clusters instead of in single files within the LVMp syncytia in LVMp´s tey mutants. However, it seems from the figure (Fig.4) that mutant LVMp syncytia contains lees number of nuclei than controls. Is this the case? I think that number of nuclei should be analysed as there might be a problem in the process of fusion with the fusion-competent myoblasts, which could affect the migratory process.
4. The authors state that in the absence of tey function, M12 founders and precursors fail to undergo dorsal migration and remain in the vicinity of the Tey-Caps+ precursors, where they erroneously respond to pathfinding cues for their epidermal attachment from tendon cells closer to their more ventral position. How do the authors envision this? Does M12 muscle differentiate properly? Often, muscles with altered orientation, which attach to ectopic muscle attachment sites, display a branched morphology with multiple attachment sites. Is this observed in the tey mutants? In Fig.7B, some branching at the anterior end of muscle M12 is observed, is this a frequent phenotype? In addition, as muscles use filopodia to search for their target tendon cells, is the dynamic of filopodia at the end of muscle M12 different in the mutant compared to controls?
5. Abnormal muscle attachment of even just one muscle has been shown to impair the movement of larvae. Do tey mutant larvae display any locomotion defects?
6. To try to get more insight into tey function, the authors could analyse the consequences of expressing an RNAi against the paralog of tey, CG4813, in LVMp´s precursors.

Reviewer 2

Advance summary and potential significance to field

The manuscript by Manfred Frasch et al., describes a new role for the RNF220 “Tey” in directing migration and differentiation of the longitudinal visceral and somatic muscle 12 in the Drosophila embryo. Previous functional analysis of Tey indicated its contribution to motor neuron target specificity, where its expression in muscle 12 represses innervation by a motor neuron targeting the

© 2023. Published by The Company of Biologists under the terms of the Creative Commons Attribution License (https://creativecommons.org/licenses/by/4.0/).

neighboring muscle 13 (Inaki et al., 2010). This repression was shown to be induced via positive expression of Toll in muscle 12. In the present manuscript a comprehensive functional analysis of Tey in promoting the migration and alignment of the longitudinal visceral muscles (LVMs) is presented. A detailed developmental expression of Tey, as well as analysis of LOF phenotype of tey in the LVMs is demonstrated with high resolution and using various specific markers of these cells. Through the creation of additional mutants in tey its contribution to the arrangement of LVMs is demonstrated throughout development.

Additional phenotypic analysis is provided to reveal the specific role of tey in directing the migration and attachment of muscle 12. It is not clear, however whether Tey function in the LVMs and M12 is mediated by Toll inhibition. The figures are impressively clear in their resolution and clarity, however the manuscript is rather descriptive and lacks further molecular insight as to how Tey exerts its function in the LVMs. One possibility that was not addressed is that Tey promotes the adhesion between the longitudinal muscles (either LVMs or muscle 12) at their longitudinal edges, and this prevents their binding to available tendon cells at the segmental border.

Comments for the author

The manuscript describes a very interesting phenotype of the LVMs, however, at its present form it lacks a deeper insight as to the molecular basis underlying the phenotype.

Reviewer 3

Advance summary and potential significance to field

In this manuscript Frasch and colleagues investigate the migration of the longitudinal visceral myoblasts of Drosophila embryos that originate in the caudal mesoderm and need to migrate over the forming midgut where they then extend longitudinally to regularly cover the gut at the mature stage. The authors characterise the late stages of this migration/elongation process in new detail. Beautiful imaging shows that the longitudinal visceral myoblasts fuse to myotubes at defined dorsal and ventral locations after their anterior migration on top of the forming circular visceral muscles that form to surround the gut. Concomitant with myoblast (or possibly myotube-myotube) fusion, the myotubes elongate longitudinally to equally cover the gut before the folding of the gut begins. The results are muscle fibers with an amazingly long aspect ratio that are extremely well oriented. A mechanism of contact inhibition is discussed. Such a detailed description of the morphogenesis of these visceral longitudinal muscles was missing from the literature and will be inspiring for future works.

Interestingly, the authors found that the nuclear factor Tey, that was formerly known because of an enhancer trap that labels a single somatic muscle (5053GAL4 labelling muscle VL1) is expressed in all the precursors of the visceral longitudinal myoblasts prior to their migration in the caudal mesoderm and is still expressed in the visceral longitudinal myotubes and myofibers at the later stages when they elongate and align along the gut. The Tey protein is localised in the nuclei, but a fuction there was unknown.

To test for function, the authors generated CRISPR based null alleles of tey (in addition to the existing lof tey[5053-GAL4] insertion. These mutants show that tey is not required for the initial long-distance migration of the longitudinal muscle precursors over the midgut, but for their later longitudinal elongation (and possible cell-cell attachment) to form thin and long longitudinal muscle fibers with an equal distribution.

Similarly, tey it is needed for VL1 founder myoblast migration to position it dorsally of the other ventral muscles. This results in abnormal morphology of the VL1 muscle in late stage tey mutants.

Overall, this is a beautifully illustrated study about 2 different muscle types of the fly, which identifies a function of tey for their normal morphogenesis. All experiments are well presented and well controlled. The conclusions are novel and supported by the data. Hence, I recommend publication.

© 2023. Published by The Company of Biologists under the terms of the Creative Commons Attribution License (https://creativecommons.org/licenses/by/4.0/).

Comments for the author

Minor points.
1. Are the elongated longitudinal muscle fibers really reaching along the entire gut at late stage 16 (line 207)
or do they form muscle-muscle attachments that would also be consistent with maintaining tension during muscle contraction when sarcomeres have formed? The latter appears to be suggested in the discussion.
2. Any integrin staining or high magnification of these fiber-fiber attachment structures would be a nice add-
on of the manuscript. Is the establishment of this attachment prior to sarcomerogenesis?

First revision

Author response to reviewers' comments

We appreciate the feed-back from the reviewers and their assessment that our manuscript presents an interesting and well-crafted study of the roles of a new player, *tey*, in muscle precursor migration. Although we do acknowledge that at this point the molecular details of *tey* function during cell migration are still unknown, we believe that the publication of its developmental functions will be of great interest and will encourage future studies on the pertinent molecular pathways and on this migration process in general.

Below please find our point-by-point response to the specific comments of the reviewers.

Reviewer 1

1. The authors claim that the LVMp's in tey mutants frequently adopt oblique or even transverse orientations, and their distances from one another are uneven. How frequent is this phenotype? I think the authors should quantify these two phenotypes, wrong orientation and aberrant distance between each other.

As suggested by the reviewer, we have carefully quantified these two phenotypes and have added the results to Fig. 4.
Fig. 4H shows violin plots depicting the distribution of the angles (compared to the A/P orientation of the midgut) of LVMp's at late st. 14 to st. 15 in control vs. *tey* mutant embryos. It shows very clearly that in the mutants there is a wide distribution of their angles from parallel to almost 90°, whereas in the controls the large majority of LVMp's deviate maximally by about 15° from the A/P axis of the midgut. Likewise, Fig. 4I shows the distance distributions of LVMp's from their nearest neighbors in *tey* mutant vs. control embryos at the same stages as above. For this purpose, we made three measurements along each LVMP, two at the ends and one in the middle. Again, it becomes clear that in the controls the muscle distances lie in a rather narrow range and they do not get too close to one another, whereas in the mutants their distances are spread over a much larger range, and frequently there are direct contacts. (Note: each dot in H and I represents a single measurement). Altogether, we think these new data nicely bolster our conclusions and we thank the reviewer for proposing this analysis.

2. The authors state that filopodia are still present in the mutant LVMp's but they have not quantified their dynamic properties. I believe that analysing in more detail some aspects of the dynamic behaviour of this filopodia in both mutant and forced overexpression contexts may help them to get some insight into possible mechanisms by which Tey might regulate muscle cell migration.

We agree that, in theory, quantifications of the dynamic properties of the filopodia could be interesting. In practice, however, this type of analysis would involve lengthy live imaging

© 2023. Published by The Company of Biologists under the terms of the Creative Commons Attribution License (https://creativecommons.org/licenses/by/4.0/).

experiments and technically the subsequent quantitative and convincing evaluation of filopodia dynamics would be extremely difficult. Hence, we decided that for the purpose of this manuscript, the time, expense, and risk of failure involved in such an analysis would not be justified.

3. The authors claim that the nuclei tend to be arranged in clusters instead of in single files within the LVMp syncytia in LVMp´s tey mutants. However, it seems from the figure (Fig.4) that mutant LVMp syncytia contains lees number of nuclei than controls. Is this the case? I think that number of nuclei should be analysed as there might be a problem in the process of fusion with the fusion-competent myoblasts, which could affect the migratory process.

We followed the suggestion of the reviewer and counted the numbers of nuclei in *tey* mutant vs. control embryos during stage 14, when myoblast fusion is still ongoing and some syncytia have already reached their full complement of 6 – 7 nuclei. These data are now shown as Fig. 4J. As can be seen, and in line with our previous visual inspection, myoblast fusion is not affected in *tey* mutant embryos.

4. The authors state that in the absence of tey function, M12 founders and precursors fail to undergo dorsal migration and remain in the vicinity of the Tey-Caps+ precursors, where they erroneously respond to pathfinding cues for their epidermal attachment from tendon cells closer to their more ventral position.

How do the authors envision this? Does M12 muscle differentiate properly? Often, muscles with altered orientation, which attach to ectopic muscle attachment sites, display a branched morphology with multiple attachment sites. Is this observed in the tey mutants? In Fig.7B, some branching at the anterior end of muscle M12 is observed, is this a frequent phenotype? In addition, as muscles use filopodia to search for their target tendon cells, is the dynamic of filopodia at the end of muscle M12 different in the mutant compared to controls?

The striations and normal shapes of M12 muscles suggest that they differentiate normally, although we cannot exclude other specific differentiation defects. As the reviewer points out, we do see examples of “split (Fig. 7D) or broadened (Fig. 7F) attachments” of M12 muscles (see lines 332-333), which we interpret such “that muscle attachments at their more ventral positions cannot be established as consistently as at their normal positions.“ (lines 394-396). So this is entirely in line with the observation of the reviewer. We have not quantified this phenotype because of the large number of tedious filet preparations that would be required, and because we did not think that quantification would add much insight in addition to the fact that these phenotypes do occur regularly. (With regard to the filopodia, please see 2. above).

5. Abnormal muscle attachment of even just one muscle has been shown to impair the movement of larvae. Do tey mutant larvae display any locomotion defects?

Locomotion defects, if any would be observed, could equally be due to the published role of *tey* in neuromuscular axon pathfinding. Because of the impossibility to derive any firm conclusion in this regard we have not monitored any locomotion defects.

6. To try to get more insight into tey function, the authors could analyse the consequences of expressing an RNAi against the paralog of tey, CG4813, in LVMp´s precursors.

We followed the suggestion of the reviewer and knocked down the *tey* paralog, *CG4813*, in LVMp’s as well as in muscle M12 precursors using *tey-GAL4* and *UAS*-dsRNA. We also included *CG13001*, which encodes a protein known to interact with CG4813 and conceivably may interact with Tey as well (as discussed in the manuscript, the mouse ortholog of CG4813, RNF220, also partners with the mouse ortholog of CG13001). As a positive control, we performed analogous knock-down experiments with *tey*. As shown in the newly added Fig. S4 and described in the text, the *tey* RNAi knock-down phenotypes of the LVMp’s and LVMu’s closely resemble those shown for various *tey* point mutants. By contrast, neither *CG4813* nor *CG13001* RNAi produced any such phenotypes. Thus, we conclude that neither *CG4813* nor *CG13001* seem to be required for proper LVMp migration, and that CG13001 is not an obligatory binding partner of Tey during this process.

© 2023. Published by The Company of Biologists under the terms of the Creative Commons Attribution License (https://creativecommons.org/licenses/by/4.0/).

Reviewer 2

One possibility that was not addressed is that Tey promotes the adhesion between the longitudinal muscles (either LVMs or muscle 12) at their longitudinal edges, and this prevents their binding to available tendon cells at the segmental border.

Regarding the LVMp's, we agree that this is a possibility that could explain why in the mutants the LVMu's fail to form the normal, long fibers. Because of the lack of space and in the absence of concrete information on this issue we did not want to speculate too much about this issue. With regard to somatic muscle M12, the adhesions as such that are formed in *tey* mutants appear normal, except that they occur at the wrong positions, as described in the manuscript.

Reviewer 3

1. Are the elongated longitudinal muscle fibers really reaching along the entire gut at late stage 16 (line 207) or do they form muscle-muscle attachments that would also be consistent with maintaining tension during muscle contraction when sarcomeres have formed? The latter appears to be suggested in the discussion.

The notion that the longitudinal muscle fibers are reaching along much of the midgut at late stage 15 and in larvae is based on the data from clonal analyses from Robert Klapper (Klapper et al., 1998), as mentioned in the text (lines 460-461). Specifically, it was found that transplantation of a single nucleus expressing lacZ can lead to beta-Gal staining of a longitudinal gut muscle along (almost) the entire length of the midgut, which suggests that ultimately it consists of a single syncytium.

2. Any integrin staining or high magnification of these fiber-fiber attachment structures would be a nice add-on of the manuscript. Is the establishment of this attachment prior to sarcomerogenesis?

As suggested by the reviewer, we did perform anti-integrin stainings to look at possible signals at LVMP interphases during their A/P alignment. However, we failed to see any signals there and did not pursue this interesting process any further, which however could be an interesting topic for future studies. (Because of the negative results and limited space we did not discuss this result and speculate more on this process, but it does occur prior to sarcomerogenesis).

Second decision letter

MS ID#: DEVELOP/2022/201457

MS TITLE: The RNF220 domain nuclear factor Teyrha-Meyrha (Tey) regulates the migration and differentiation of specific visceral and somatic muscles in Drosophila

AUTHORS: Manfred Frasch, Afshan Ismat, Ingolf Reim, and Jasmin Raufer
ARTICLE TYPE: Research Article

I am happy to tell you that your manuscript has been accepted for publication in Development, pending our standard ethics checks.

Reviewer 2

Advance summary and potential significance to field

The manuscript by Manfred Frasch et al., describes a new role for the RNF220 "Tey"

© 2023. Published by The Company of Biologists under the terms of the Creative Commons Attribution License (https://creativecommons.org/licenses/by/4.0/).

in directing migration and differentiation of the longitudinal visceral and somatic muscle 12 in the Drosophila embryo. Although the manuscript is rather descriptive and lacks further molecular insight as to how Tey exerts its function in the LVMs it is valuable in that it describes a novel function for TEY.

Comments for the author

I have no further requests.

Reviewer 3

Advance summary and potential significance to field

As stated at length in my initial review, I find this manuscript by Frasch and co-workers and exciting as it details the morphogenesis of an important part of the larval musculature, the longitudinal visceral muscles of the fly larvae. The identification of Tey in the process provides a first handle in how these muscles are specified and instructed for during morphogenesis. Imaging is beautiful and genetics shows the Tey function beyond any doubt.

Comments for the author

All my minor points were addressed in the revised version and the manuscript has been further improved by helpful suggestions from the other reviewers, mainly on quantifying the orientation of the mutant LVMs better. All additional experiments strongly support the initial conclusions of the manuscript. Thus, I strongly support publication.

© 2023. Published by The Company of Biologists under the terms of the Creative Commons Attribution License (https://creativecommons.org/licenses/by/4.0/).
